# *cis*‐prenyltransferase 3 and α/β‐hydrolase are new determinants of dolichol accumulation in Arabidopsis

**DOI:** 10.1111/pce.14223

**Published:** 2021-11-29

**Authors:** Katarzyna Gawarecka, Joanna Siwinska, Jaroslaw Poznanski, Agnieszka Onysk, Przemyslaw Surowiecki, Karolina Sztompka, Liliana Surmacz, Ji Hoon Ahn, Arthur Korte, Ewa Swiezewska, Anna Ihnatowicz

**Affiliations:** ^1^ Institute of Biochemistry and Biophysics Polish Academy of Sciences Warszawa Poland; ^2^ Department of Life Sciences Korea University Seoul Korea; ^3^ Intercollegiate Faculty of Biotechnology of University of Gdansk and Medical University of Gdansk University of Gdansk Gdansk Poland; ^4^ Center for Computational and Theoretical Biology University of Wurzburg Wurzburg Germany

**Keywords:** dolichol, GWAS, isoprenoid, natural variation, plant‐environment interactions, polyprenol, QTL mapping, secondary metabolism

## Abstract

Dolichols (Dols), ubiquitous components of living organisms, are indispensable for cell survival. In plants, as well as other eukaryotes, Dols are crucial for post‐translational protein glycosylation, aberration of which leads to fatal metabolic disorders in humans and male sterility in plants. Until now, the mechanisms underlying Dol accumulation remain elusive. In this study, we have analysed the natural variation of the accumulation of Dols and six other isoprenoids among more than 120 *Arabidopsis thaliana* accessions. Subsequently, by combining QTL and GWAS approaches, we have identified several candidate genes involved in the accumulation of Dols, polyprenols, plastoquinone and phytosterols. The role of two genes implicated in the accumulation of major Dols in Arabidopsis—the AT2G17570 gene encoding a long searched for *cis*‐prenyltransferase (CPT3) and the AT1G52460 gene encoding an α/β‐hydrolase—is experimentally confirmed. These data will help to generate Dol‐enriched plants which might serve as a remedy for Dol‐deficiency in humans.

## Summary Statement

The accumulation of Dolichols in Arabidopsis is affected by the cis‐prenyltransferase 3 (CPT3) and an α/β‐hydrolase (ABH). Using QTL and GWAS approaches we have identified these two and several other candidate genes involved in the accumulation of Dols, polyprenols, plastoquinone and phytosterols.

## 1 INTRODUCTION

**Figure 1:**
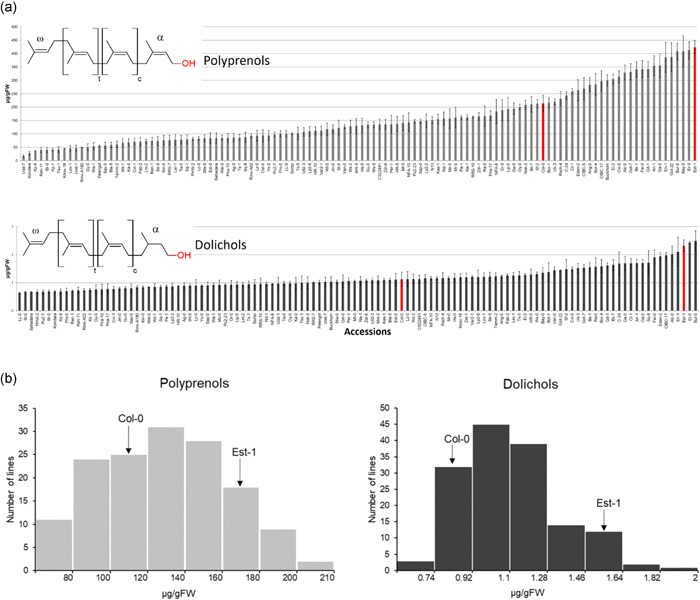
Polyisoprenoid lipids of *Arabidopsis thaliana*. (a) Content of polyprenols (Pren) and dolichols (Dols) in Arabidopsis accessions. Bars representing Col‐0 and Est‐1 are marked in red. Shown are means ± SD (*n* = 3). Content of other isoprenoids (chlorophylls, carotenoids, phytosterols, plastoquinone and tocopherols) in the seedlings of Arabidopsis accessions are given in Figure S3. (b) Frequency distribution of the content of polyprenols and Dols in the seedlings of AI‐RILs and their parental lines, Col‐0 and Est‐1. Each bar covers the indicated range of a particular isoprenoid compound. Frequency distribution of the content of other isoprenoids (chlorophylls, carotenoids, phytosterols, plastoquinone and tocopherols) are given in Figure S4. Structures of polyprenol and dolichol are shown in the inset: t and c stand for the number of internal isoprene units in *trans* and *cis* configuration, respectively. The α‐ and ω‐terminal isoprene units are depicted [Color figure can be viewed at wileyonlinelibrary.com]

Isoprenoids (also known as terpenes) are a large and diverse group of compounds comprised of more than 40 000 chemical structures (Bohlmann & Keeling, 2008). Linear polymers containing from 5 to more than 100 isoprene units are called polyisoprenoids (Swiezewska & Danikiewicz, 2005). Due to the hydrogenation status of their OH‐terminal, (α‐) isoprene unit, polyisoprenoids are subdivided into α‐unsaturated polyprenols (hereafter named Prens) and α‐saturated dolichols (hereafter named Dols) (Figure 1). Prens are common for bacteria, green parts of plants, wood, seeds and flowers, while Dols are constituents of plant roots as well as animal and fungal cells (Rezanka & Votruba, 2001). In eukaryotic cells, the dominating polyisoprenoid components are accompanied by traces of their counterparts, for example, Prens are accompanied by Dols in photosynthetic tissues (Skorupinska‐Tudek et al., 2003).

All isoprenoids are synthesised from isopentenyl and dimethylallyl diphosphate (IPP and DMAPP) molecules, which in plants are derived from the cytoplasmic mevalonate (MVA) and plastidial methylerythritol phosphate (MEP) pathways (Hemmerlin et al., 2012; Lipko & Swiezewska, 2016). Formation of the polyisoprenoid chains of both Pren and Dol from IPP is executed by enzymes called *cis*‐prenyltransferases (CPTs), which are responsible for elongation of an all‐*trans* initiator molecule, most commonly farnesyl or geranylgeranyl diphosphate. This reaction generates a mixture of polyprenyl diphosphates (PolyprenylPP) of similar, CPT‐specific, lengths. In *Arabidopsis thaliana* (hereafter named Arabidopsis), only three (Akhtar et al., 2017; Cunillera et al., 2000; Kera et al., 2012; Oh et al., 2000; Surmacz et al., 2014; Surowiecki et al., 2019) out of nine putative CPTs (Surmacz & Swiezewska, 2011) have been characterised at the molecular level. Interestingly, none of these well‐characterised CPTs (CPT1, ‐6 or ‐7) is responsible for the synthesis of the major ‘family’ of Dols (Dol‐16 dominating) accumulated in Arabidopsis tissues. The polyprenyl diphosphates resulting from CPT activity undergo then either dephosphorylation to Prens and/or reduction to Dols. The reduction reaction is catalysed by polyprenol reductases, two of which have been recently described in Arabidopsis (Jozwiak et al., 2015). Although this biosynthetic scheme is generally accepted some steps of Pren and Dol biosynthesis pathways remain unknown. A simplified scheme depicting main steps leading to formation of Prens, Dols as well as other isoprenoid compounds analysed in this report is presented in Figure S1.

Isoprenoids are implicated in vital processes in plants, for example, in photosynthesis and stress response (chlorophylls, carotenoids, plastoquinone and tocopherols), or in the synthesis of plant hormones (carotenoids and sterols), or they function as structural components of membranes (sterols) (Tholl, 2015). Polyisoprenoids are modulators of the physico‐chemical properties of membranes, but they are also involved in other specific processes. Dolichyl phosphate (DolP) serves as an obligate cofactor for protein glycosylation and for the formation of glycosylphosphatidylinositol (GPI) anchors, while Prens, in turn, have been shown to play a role in plant photosynthetic performance (Akhtar et al., 2017). Importantly, an increased content of Prens improves the environmental fitness of plants (Hallahan & Keiper‐Hrynko, 2006). Additionally, it has also been suggested that in plants Prens and Dols might participate in cell response to stress since their content is modulated by the availability of nutrients (Jozwiak et al., 2013) and by other environmental factors (xenobiotics, pathogens and light intensity) (summarised in Surmacz & Swiezewska, 2011). Moreover, the cellular concentration of Prens and Dols is also considerably increased upon senescence (summarised in Swiezewska & Danikiewicz, 2005). These observations suggest that eukaryotes might possess, so far elusive, regulatory mechanisms allowing them to control polyisoprenoid synthesis and/or degradation.

Most traits important in agriculture, medicine, ecology and evolution, including variation in chemical compound production, are of a quantitative nature and are usually due to multiple segregating loci (Mackay, 2001). Arabidopsis is an excellent model for studying natural variation due to its genetic adaptation to different natural habitats and its extensive variation in morphology, metabolism and growth (Alonso‐Blanco et al., 2009; Fusari et al., 2017). Natural variation for many traits has been reported in Arabidopsis, including primary and secondary metabolism (Keurentjes et al., 2006; Kliebenstein et al., 2001; Lisec et al., 2008; Meyer et al., 2007; Mitchell‐Olds & Pedersen, 1998; Rowe et al., 2008; Sergeeva et al., 2004; Tholl et al., 2005; Siwinska et al., 2014). Until now, no systematic analysis of the natural variation of polyisoprenoids has been performed for any plant species.

Therefore, in this study, we decided to use the model plant Arabidopsis to explore the natural variation of Prens and Dols. Importantly, Arabidopsis provides the largest and best‐described body of data on the natural variation of genomic features of any plant species (Kawakatsu et al., 2016; The 1001 Genomes Consortium, 2016). Over 6000 different Arabidopsis accessions that can acclimate to enormously different environments (Kramer, 2015) have been described so far (Weigel & Mott, 2009).

To identify genes that are responsible for modulation of polyisoprenoid content, we used both a quantitative trait loci (QTL) mapping approach and genome‐wide association studies (GWAS). So far, neither QTL nor GWAS has been used for the analysis of Prens and Dols. Traditional linkage mapping usually results in detection of several QTLs with a high statistical power, making it a powerful method in the identification of genomic regions that co‐segregate with a given trait in mapping populations (Koornneef et al., 2004; Korte & Farlow, 2013). But the whole procedure including the identification of underlying genes is usually time‐consuming and laborious. GWAS studies profit from a wide allelic diversity, high resolution and may lead to the identification of more evolutionarily relevant variation (Kooke et al., 2016). It is possible to overcome some limitations of QTL analyses by using the GWAS approach, which can be used to narrow down the candidate regions (Han et al., 2018; Korte & Farlow, 2013). But it should be kept in mind that GWAS also has its limitations, such as dependence on the population structure, the reliance on SNPs rather than gene structural variants or the potential for false‐positive and false‐negative errors (Korte & Farlow, 2013; Zhu et al., 2008). We have applied here both QTL mapping and GWAS analyses because it has been shown that the combination of these two methods can alleviate their respective limitations (Brachi et al., 2010; Zhao et al., 2007).

The application of QTL and GWAS described here led to identification of several candidate genes underlying the accumulation of polyisoprenoids. Additionally, to get insight into the biosynthetic pathways of Dols and Prens in a broader cellular context, a set of seven isoprenoid compounds was analysed and subsequently candidate genes were selected. The most interesting of the identified genes were *cis‐prenyltransferase 3* (*CPT3*, AT2G17570, identified through QTL mapping) and *α/β‐hydrolase* (*ABH*, AT1G52460, identified through GWAS). CPT3, although biochemically not characterised, has been demonstrated to efficiently incorporate in vitro IPP into *cis*‐polyisoprenoid of an undefined chain‐length thus to possess a CPT‐like activity; moreover, its expression complemented the yCTP deficiency (Kwon et al., 2016), whereas ABH has not been previously connected with polyisoprenoid biosynthesis. In this study, their involvement in Dol biosynthesis/accumulation is experimentally confirmed using mutant approach, metabolite profiling, yeast transformation, transient expression in *Nicotiana benthamiana* leaves, bimolecular fluorescence complementation (BiFC) and yeast two‐hybrid (Y2H) assays. Although obtained results clearly suggest the role of CPT3 and ABH in Dol accumulation one should remember that in this report analysis of the level of terpene was limited to the seedling stage and might differ for mature plants. Moreover, it should be kept in mind, however, that although CPT3 and ABH, together with other genes depicted in this report, are strong candidates for being causal for the observed natural variation more studies are required to prove such role. Importantly, identification of CPT3 fills the gap in the Dol biosynthetic route in Arabidopsis and, together with newly depicted ABH, makes the manipulation of Dol content in plants feasible. Consequently, an option for the generation of plant tissues with increased Dol content as dietary supplements for individuals suffering from Dol‐deficiency is emerging. Moreover, presuming conserved role of ABH in Dol pathway in eukaryotes a design of a new therapeutic strategy ameliorating Dol deficiency via manipulation of the activity of respective human ABH seems plausible.

## 2 MATERIALS AND METHODS

### 2.1 Plant materials

*A. thaliana* accessions used in this study are listed in the Supporting Information (Table S9). All accessions were obtained from the stock center NASC (http://arabidopsis.info/).

A population of advanced intercross recombinant inbred lines (AI‐RIL and EstC) was obtained after crossing of the Est‐1 (Estland) and Col‐0 (Columbia) accessions (Balasubramanian et al., 2009). All lines were kindly provided by Maarten Koornneef from Max Planck Institute for Plant Breeding Research in Cologne, Germany. The EstC mapping population together with the marker data are available at the NASC under the stock number CS39389.

The seeds of T‐DNA insertion mutant lines for AT1G52460, SALK_066806 and GK_823G12, were obtained from the Nottingham Arabidopsis Stock Center, their progeny was genotyped, and heterozygous lines were isolated.

### 2.2 Generation of *CPT3* RNAi and *CPT3*‐over‐expressing lines

For miRNA‐mediated knockdown of the *CPT3* gene, two pairs of primers specific to amiRNA and amiRNA* targeting the gene were designed using the Web MicroRNA Designer WMD3. The vector pRS300 was used as a template for subsequent PCR amplification and replacement of the endogenous miR319a and miR319a* sequences with appropriate amiRNA and amiRNA* of *CPT3* as described in the website protocol wmd3.weigelworld.org (Ossowski Stephan, Fitz Joffrey, Schwab Rebecca, Riester Markus and Weigel Detlef, personal communication). The obtained stem‐loop was used as a template for PCR to generate the 454 bp fragment with a CACC overhang at the 5′ end, which was used for directional cloning into the pENTR/D‐TOPO vector system (Invitrogen). The recombination reaction from pENTR/D‐TOPO to the pGWB602 binary vector was carried out with the Gateway LR clonase II system (Invitrogen). All primers used in the construction of the *CPT3* silencing vector are listed in Table S10. The obtained plasmid was introduced into *Agrobacterium tumefaciens* strain GV3101, which was then used to transform Arabidopsis (Col‐0) by the floral dip method (Weigel & Glazebrook, 2002). T1 seeds were germinated on soil and transgenic plants were selected by spraying with 0.1% BASTA in the greenhouse. Spraying was performed 1 week after germination and was repeated two times at 2‐day intervals. Additionally, the plants that survived were verified by PCR.

*CPT3*‐over‐expressing lines (*CPT3‐OE*) were generated using a 35S::*CPT3* construct introduced into the *A. tumefaciens* GV3101 strain. Transformation of Arabidopsis (Col‐0) plants was performed by the floral dip method (Weigel & Glazebrook, 2002). Transformant selection was performed as described previously (Surowiecki et al., 2019).

### 2.3 Growth conditions

Plants were grown in a growth chamber in a long day (16‐h light) photoperiod at 22°C/18°C at day/night. The seeds were surface‐sterilised by treatment with an aqueous solution of 5% calcium hypochlorite for 8 min, subsequently rinsed four times with sterile water and planted on plates. Before location in the growth chamber, plates with seeds were kept for 4 days at 4°C in darkness for stratification. The Arabidopsis accessions and the AI‐RIL mapping population dedicated for metabolite profiling were grown on large (150 diameter) Petri dishes on solid ½ Murashige‐Skoog medium with vitamins (1 L of medium contained 0.5 µg nicotinic acid, 0.5 µg pyridoxine, 0.1 µg thiamine, 2 µg glycine) and 0.8% agar. One plate was used as one biological replicate (*n* ≈ 50–100 plants), at least three biological replicates were used for metabolite profiling. T‐DNA insertion mutant lines used for genotyping and RNA were cultivated in soil mixes in at least three biological replicates.

### 2.4 Isolation of isoprenoids

Unless indicated otherwise, entire 3‐week‐old seedlings were used for the isolation of all isoprenoid compounds. Plants from each individual Petri dish were subdivided into four aliquots, weighed and subjected to four different extraction methods dedicated to the isolation of prenols, Dols and sterols (3 g); tocopherols (3 g); plastoquinone (0.5 g) and chlorophyll and carotenoids (0.2 g). The size differences among the used Arabidopsis accessions grown on MS plates after 3 weeks of cultivation were negligible. After this short time, all accessions were in the phase of vegetative growth.

To elucidate the correlation between polyisoprenoid content versus *CPT3* transcript level, the Arabidopsis seedlings, leaves and flowers were used. For qualitative and quantitative analysis of isoprenoids, either internal (Prens, Dols and phytosterols) or external (plastoquinone and tocopherol) standards were employed. For quantitative analysis of Prens, Dols, phytosterols, plastoquinone and tocopherols signals corresponding to compounds of well‐characterised structure were taken into consideration, exclusively.

Prens, Dols, phytosterols, plastoquinone, tocopherols, carotenoids and chlorophylls were isolated and quantified using standard methods—for details see Supporting Information.

### 2.5 Complementation of the yeast *rer2Δ* mutant

To express *CPT3* and *LEW1* in *Saccharomyces cerevisiae* mutant cells (*rer2*Δ mutant: *rer2*::kanMX4 *ade2‐101 ura3‐52 his3‐200 lys2‐801*), coding sequences of *CPT3* and *LEW1* (AT1G11755) were subcloned into the pESC‐URA yeast dual expression vector (Agilen) according to the manufacturer's protocol. Transformant selection and growth, as well as analyses of polyisoprenoid profile and CPY glycosylation status, were performed as described previously (Surowiecki et al., 2019).

### 2.6 Subcellular localisation and BiFC assays

For subcellular localisation analysis of *35S::CPT3, A. tumefaciens* cells carrying the vectors CPT3‐GFP and cd3‐954 (ER‐CFP, used as an organelle marker) were introduced into the abaxial side of *N. benthamiana* leaves. A BiFC assay was performed based on split EYFP. EYFP was fused to the C‐terminus of CPT3 and the N‐terminus of Lew1, resulting in the expression of CPT3:EYFPC and Lew1:EYFPN. CPT3:EYFPC was co‐infiltrated with Lew1:EYFPN into the abaxial side of *N. benthamiana* leaves. A positive fluorescence signal (EYFP) is indicative of the restoration of EYFP due to the heterodimerization of CPT3 with Lew1.

The transient expression of CPT3, ER‐CFP and CPT3/Lew1‐YFP fusion proteins was observed under a Nikon C1 confocal system built on TE2000E with 408, 488 and 543 nm laser excitations for CFP (450/35 nm emission filter) and GFP (515/30 nm emission filter), respectively.

### 2.7 Y2H assay

To test protein‐protein interactions coding sequences of CPT3 and LEW1 were subcloned into the pENTR/D‐TOPO vector and next recombined into Y2H vectors (pGADT7‐GW and pGBKT7‐GW) using LR Clonase II. Selected constructs were transformed into *S. cerevisiae* AH109 strain (MATa, trp 1—901, leu2—3, 112, ura 3—52, his3—200, gal4△, gal80△, LYS2: GAL1UAS—GAL1TATA—HIS3, GAL2UAS—GAL2TATA—ADE2, URA3: MEL1UAS—MEL1TATA—LacZ) using the lithium acetate method. Double transformant colonies selected by colony PCR were grown in media lacking leucine and tryptophan (‐Leu/‐Trp). Serial dilutions of the selected double transformants were grown in plates lacking leucine, tryptophan and histidine (‐Leu/‐Trp/‐His) supplemented with 1 mM 3‐AT (3‐Amino‐1,2,4‐triazole). The experiments were performed in at least three replicates.

Y2H vectors pGADT7‐GW (Addgene plasmid #61702; http://n2t.net/addgene:61702; RRID:Addgene_61702) and pGBKT7‐GW (Addgene plasmid #61703; http://n2t.net/addgene:61703; RRID:Addgene_61703) were a gift from Yuhai Cui (Lu et al., 2010).

### 2.8 Statistical analyses

#### 2.8.1 Quantitative genetic analyses

Mean values of at least three replicates were calculated for each isoprenoid compound measured, for each AI‐RIL and each natural accession. These values were used in QTL mapping and GWAS. The broad sense heritability (H^2^) for isoprenoid accumulation for the AI‐RIL population was estimated according to the formula: *H* ^2^ = *V*_G_/(*V*_G_ + *V*_E_), where *V*_G_ is the among‐genotype variance component and *V*_E_ is the residual (error) variance. For GWAS heritability, estimates have been extracted from the mixed model accordingly.

#### 2.8.2 QTL analyses in the AI‐RIL population

All obtained phenotypical data were used in QTL mapping that was performed using R software (R Core Team, 2016; https://www.R-project.org/) with R/qtl package (Arends et al., 2010; Broman et al., 2003; http://www.rqtl.org/). Stepwise qtl function was used to detect multiple‐QTL models (Broman, 2008; http://www.rqtl.org/tutorials/new_multiqtl.pdf). This function requires single‐QTL genome scan to locate QTLs with the highest LOD scores, then the initial model is tested using arguments for additional QTLs and interactions between QTLs search, model refinement and backward elimination of each QTL detected back to the null model. Significance threshold (LOD) value (*p* < 0.05) for this mapping population of plants was established from 10 000 permutations to 3.4. Obtained QTL models were refined with the refineqtl function; any possible interactions between QTLs were verified by the addint function. See Table S2 for detailed description of the procedure of selection of candidate genes from chosen QTL intervals.

#### 2.8.3 GWAS

Genome‐wide association mapping was performed on measurements for 115–119 different natural accessions per phenotype. The phenotypic data are available at the AraPheno database (Seren et al., 2016) and the mean phenotypic values per accession have been used for GWAS. Eighty‐six of these lines have been recently sequenced as part of the 1001 genomes project and full sequence information is readily available (1001 Genomes Consortium, 2016). For the remaining accessions, high‐density SNP data have been published earlier (Horton et al., 2012). The genotypic data for all 119 accessions used have been generated by imputing the missing SNP calls (as described in Togninalli et al., 2018) and contain 4 314 718 SNPs. Around two million of these polymorphisms had a minor allele count of at least five and were included in the analysis.

GWAS was performed with a mixed model correcting for population structure in a two‐step procedure, where first all polymorphisms were analysed with a fast approximation (emmaX, Kang et al., 2010) and afterwards the top 1000 polymorphisms were reanalysed with the correct full model. The kinship matrix has been calculated under the assumption of the infinitesimal model using all sequence variants with a minor allele frequency of more than 5% in the whole population. The analysis was performed in R (R Core Team, 2016). The R scripts used are available at https://github.com/arthurkorte/GWAS. The Bonferroni‐corrected 5% significance threshold for the analysed markers was of 2.4 × 10^−8^. Power for GWAS was calculated using the *pwr.p.test* function implemented in the R package *pwr* (R Development Core Team 2008).

To assess the genetic correlation between the different traits, a multi‐trait mixed model (Korte et al., 2012) was used that estimates the amount of phenotypic variation that is caused by shared genetic factors.

#### 2.8.4 Correlation analyses of isoprenoid accumulation: A statistical meta‐analysis

All correlation analyses were performed with the aid of R version 3.3.0 (R Core Team, 2016, https://www.R-project.org/) using the outliers (Komsta, 2011; R package version 0.14, https://CRAN.R-project.org/package=outliers) and the gplots (Warnes et al., 2016; R package version 3.0.1, https://CRAN.R-project.org/package=gplots). The significance level α of 0.001 was assumed in all statistical tests.

For each accession, the level of each metabolite was measured in triplicate and the values thus obtained were analysed collectively, as indicated by the number of experimental points in the respective figures (which equals three times the number of accessions). Consequently, the means for replicates (as well as their standard errors treated as uncertainties) were not used. The proposed approach was employed to avoid the problem of adjusting and weighing mean values and to allow testing for outliers among single replicates instead of mean values, which implies removal of a given accession/metabolite datapoint. The method used makes calculated correlation coefficient less sensitive to the bias of individual measurements.

The Shapiro–Wilk test (Shapiro & Wilk, 1965) was used to assess the agreement of isoprenoid content in the populations with the Gaussian distribution. Since, even after filtering out of extreme values with the Grubbs’ test for outliers (Grubbs, 1950), a vast majority of the distributions were found non‐Gaussian, further analyses were performed using non‐parametric methods. Consequently, a correlation matrix for the seven investigated isoprenoids was calculated accordingly to the Spearman's rank correlation coefficients (Spearman, 1904).

A hierarchical cluster analysis of the correlation matrix was performed according to the Ward criterion (Ward, 1963).

### 2.9 Quantitative real‐time PCR analysis

Total RNA from Col‐0, Stw‐0 and Or‐0 seedlings (1‐, 2‐ and 3‐week‐old) and leaves (4‐, 5‐ and 6‐week‐old plants) was isolated and purified using RNeasy Plant Mini Kit (Qiagen) following the manufacturer's instructions. RNA concentration and purity were verified using a NanoDrop™ 1000 Spectrophotometr (Thermo Scientific). RNA was treated with RNase‐free DNase I (Thermo Scientific) according to the manufacturer's instructions. One hundred and sixty nano gram RNA per each sample was used for first‐strand synthesis using SuperScript™ II First‐Strand Synthesis System for RT‐PCR (Thermo Scientific) and oligo‐dT primers according to the manufacturer's procedure. Two microliter of cDNA was used for real‐time PCR analysis, using 0.6 µl each of gene‐specific primers listed in Table S10 in a total volume of 20 µl of Luminaris HiGreen High ROX qPCR Master Mix (Thermo Scientific) in a real‐time thermal cycler STEPOnePlus (A&B Applied Biosystems) as instructed. Statistical analysis was performed using Annova with a post‐hoc Tukey test.

## 3 RESULTS

### 3.1 Phenotypic variation in isoprenoid content among Arabidopsis accessions

A set of 116 natural Arabidopsis accessions, originating from various geographical locations, was carefully selected for a detailed analysis of seven isoprenoid compounds (carotenoids, chlorophylls, Dols, phytosterols, plastoquinone, Prens and tocopherols). Levels of seven selected isoprenoids were quantified in 3‐week‐old seedlings grown on solid Murashige‐Skoog medium. For all analysed accessions, the same types of isoprenoids were observed, however, their level differed remarkably. Thus, for all accessions, one ‘family’ of Prens composed of 9–12 isoprene units (Pren‐9 to ‐12, Pren‐10 dominating) and one ‘family’ of Dols (Dol‐15 to ‐18, Dol‐16 dominating) were detected (HPLC/UV, Figure S2a); however, the content of Prens and Dols revealed remarkable variation between accessions (Figure 1a). The highest difference in Pren content was observed for the accessions Est‐1 and Uod‐7 (20‐fold), while in Dol content—for LL‐0 and Bur‐0 (4‐fold). Similar observations were noted for the remaining isoprenoids—although the profile was the same for all accessions (Figure S2b–d), their content revealed substantial differences (Figure S3a–e). For phytosterols—5‐fold (Sav‐0 vs. Est‐1), for plastoquinone—25‐fold (Mr‐0 vs. Er‐0), for tocopherols—8‐fold (Lip‐0 vs. Edi‐0), for carotenoids—4‐fold (Est‐1 vs. CS22491) and for chlorophylls—5‐fold (Br‐0 vs. CS22491). Detailed analyses revealed considerable differences in the content of 5 out of 7 analysed compounds (i.e., Prens, Dols, phytosterols, carotenoids and plastoquinone) between Est‐1 and Col‐0 (Figures 1a and S3a–e).

Moreover, Est‐1 and Col‐0 are the parents of the advanced intercross recombinant inbred lines (AI‐RILs) mapping population (EstC), which is an excellent resource for QTL analyses due to a large number of fixed recombination events and the density of polymorphisms (Balasubramanian et al., 2009). For these reasons, the EstC population was selected for further analyses in addition to the analysis of the natural accessions.

### 3.2 Phenotypic variation in isoprenoid content in the AI‐RIL mapping population

Next, the seven isoprenoid compounds described above (carotenoids, chlorophylls, Dols, phytosterols, plastoquinone, Prens and tocopherols) were quantified in 146 lines of the EstC mapping population and its parental lines (Col‐0 and Est‐1). The profiles of analysed isoprenoids were similar to those described above for different accessions, while the level of particular compounds varied among lines of the mapping population (shown in details in Figures 1b, S4, and Table 1). The range of the content of Prens (Figure 1b), Dols (Figure 1b) and other compounds (Figure S4) was broader than that observed for both parental lines, which might suggest that several loci within the EstC population contribute to this phenomenon and it may be explained by the presence of transgressive segregation.

**Table 1:** Isoprenoid content: parental values, ranges and heritabilities in the AI‐RIL mapping population (see Section 2)

Isoprenoid compound values (µg/gFW)	Parental lines		AI‐RILs		
	Col‐0	Est‐1	Range	Median (quartiles)	Heritability^a^
Prenols	116 ± 10	179 ± 5	60–209	129 (104; 153)	0.55
Dolichols	0.9 ± 0.1	1.6 ± 0.5	0.7–2.0	1.1 (0.9; 1.2)	0.55
Chlorophylls	503 ± 29	250 ± 8	222–604	392 (349; 441)	0.42
Carotenoids	125 ± 18	75 ± 8	57–140	94 (84; 104)	0.43
Phytosterols	98 ± 11	125 ± 3	74–154	107 (97; 117)	0.33
Plastoquinone	99 ± 12	148 ± 12	50–176	111 (97; 127)	0.47
Tocopherols	138 ± 34	226 ± 36	76–288	142 (121; 163)	0.57

^a^ Measure of total phenotypic variance attributable to genetic differences among genotypes (broad sense heritability) calculated as *V*_G_/(*V*_G_ + *V*_E_).

### 3.3 Estimation of the heritability of isoprenoid levels

To identify the fraction of the observed variation that is genetically determined, we estimated the broad sense heritability (*H* ^2^) for each isoprenoid (Table 1) as described in Section 2. In the AI‐RIL population, the broad sense heritability ranged from 0.33 (for Phytosterols) to 0.55 (for Pren and Dol) and 0.57 (for Tocopherols) (Table 1).

### 3.4 Identification of QTLs for the accumulation of Dols, Prens, chlorophylls and carotenoids

The collected biochemical data for the EstC mapping population were subsequently used to map QTL regions underlying the observed phenotypic variation in isoprenoid accumulation. We were able to map QTLs for four types of compounds (Prens, Dols, chlorophylls and carotenoids). We detected three QTLs on chromosome 5 for Pren accumulation (Figure S5a) (127.3–133.4, 166.5–170.8 and 171.1–173.3 cM), explaining approximately 33% of the phenotypic variance explained (PVE) by these QTLs containing 948 genes (Table S1). For Dol, we detected a QTL region on chromosome 2 (Figure S5b) (64.8–74.4 cM) containing 308 genes (Table S1), which explains approximately 16.8% of the PVE.

Two QTLs were detected for chlorophyll accumulation on chromosome 2 (160.8–191.6 cM) and 3 (111.6–188.1 cM) (Figure S5c), which together explain 16% of the PVE (Table S1). On chromosome 2, 3 and 5 (159.3–196.5, 131.3–145.6 and 151.3–187.2, respectively) (Figure S5d), we identified three QTLs underlying the variation in carotenoid accumulation, as the whole model explains together almost 24% of the PVE (Table S1). It should be underlined that the QTL on chromosome 3 (for chlorophylls) and the QTL on chromosome 5 (for carotenoids) were included in this analysis even though their LOD scores were below the threshold (below 3) (Figure S5c,d, respectively). Interestingly, two of the QTLs identified for chlorophylls and carotenoids, localised on chromosomes 2 and 3, were overlapping.

Our search also revealed two small QTL regions for phytosterols (data not shown); however, they were not analysed further due to their statistical insignificance (LOD < 3.0). Despite the large set of numerical data, no QTLs were identified for plastoquinone or tocopherols. This might indicate that the mapping population used in this study was not appropriate for investigating these metabolites.

### 3.5 Selection of candidate genes from QTL mapping

To select and prioritise positional candidate genes from the QTL confidence intervals, we conducted a literature screen and an in silico analysis (explained in more detail in Section 2) that were based on functional annotations, gene expression data and tissue distribution of the selected genes. We analysed genes from the Dol‐associated QTL (DOL1) and from the three Pren‐associated QTLs (PRE1, PRE2 and PRE3). We selected the intervals that were characterised by the highest percentage of phenotypic variance related to each QTL and the highest LOD score values linked with the lowest number of genes (Table S1). As a result of the above‐described procedure of selection and prioritisation, we generated four sets of genes—three for Prens (Table S2) and one for Dol (Table S3).

Within a set of potential candidate genes for Pren (Table S2), there was the AT5G45940 gene encoding the Nudix hydrolase 11 (Kupke et al., 2009) with putative IPP isomerase activity. For Dol biosynthesis, we identified three genes that might be directly implicated in the process: AT2G17570, encoding a *cis*‐prenyltransferase 3 (CPT3), AT2G17370, encoding HMGR2 (hydroxymethylglutaryl Coenzyme‐A reductase 2, also called HMG2, a highly regulated enzyme that constitutes a rate‐limiting step in the MVA pathway) and AT2G18620, encoding a putative GGPPS2 (geranylgeranyl diphosphate synthase 2). A brief comment on the putative role of the two latter genes in the Dol pathway is presented in Table S3, while an in‐depth characteristic of AT2G17570 (CPT3) is presented below.

### 3.6 The role of CPT3 in Dol synthesis in Arabidopsis: Genetic and biochemical studies

Remarkably, the CPT responsible for the formation of the hydrocarbon backbone of the major Dols (Dol‐15 to Dol‐17) in Arabidopsis has not been identified yet. The AT2G17570 gene encoding CPT3 (sometimes named CPT1 [Kera et al., 2012]) is ubiquitously expressed in Arabidopsis organs and, among all nine AtCPTs, it is by sequence homology the closest counterpart of the yeast CPTs that synthesize Dols (Surmacz & Swiezewska, 2011). Preliminary studies revealed that *CPT3*, when co‐expressed with *LEW1*, was capable of rescuing the growth defect of a yeast strain devoid of both yeast CPTs: *rer2Δ srt1Δ*, and a microsomal fraction of thus obtained yeast transformant was able to incorporate in vitro a radioactive precursor into polyisoprenoids, although their profile had not been presented (Kwon et al., 2016).

**Figure 2:**
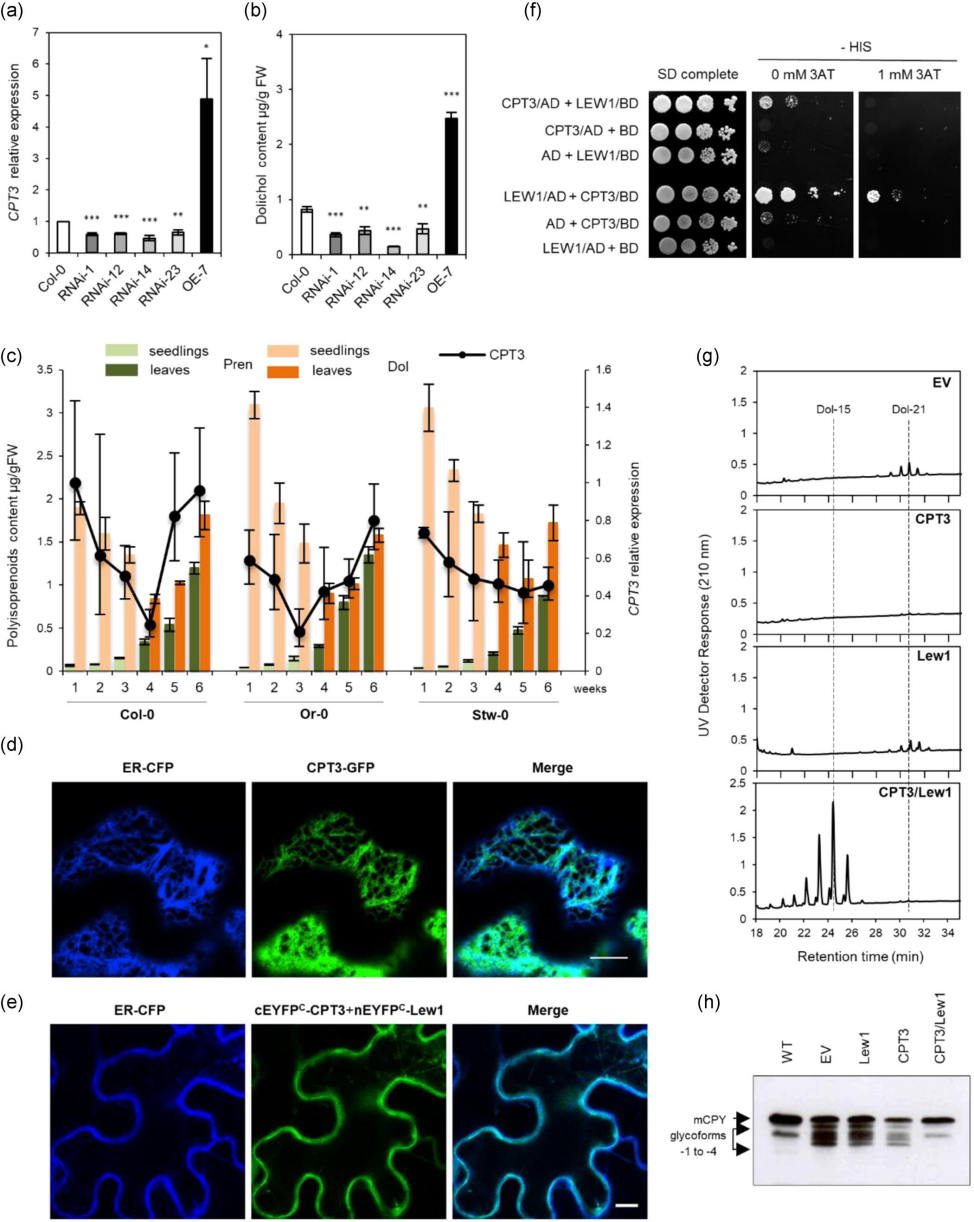
Role of CPT3 in Dol biosynthesis—studies in planta and functional assay in yeast. (a,b) Relative expression of *CPT3* and content of dolichols (Dol‐15 to Dol‐17) in the leaves of 4‐week‐old Arabidopsis plants, measured for wild‐type Col‐0, four independent RNAi lines targeting *CPT3* (RNAi‐1, ‐12, ‐14 and ‐23) and a *CPT3*‐overexpressing line (OE‐7). The results are means (±SD) of three independent experiments. Asterisks indicate statistically significant differences between WT and mutant plants (*0.01 < *p* < 0.05, **0.001 < *p* < 0.01, ***p < 0.001, Student's *t*‐test). (c) Changes in the levels of *CPT3* mRNA (black curves) and in the content of Dols and Prens (respectively, orange and green bars) in the tissues of three Arabidopsis accessions: Col‐0, Or‐0 and Stw‐0, during the plant life‐span. Transcript levels and lipid content were estimated in Arabidopsis seedlings (1–3 weeks, bright colours) and leaves (4–6 weeks, deep colours), shown are means ± SD (*n* = 3). Please note that the content of Prens is rescaled (0.01 multiples are presented) due to their high cellular level. (d) Co‐localisation of fluorescence signals of CPT3‐GFP (green) and ER‐CFP (compartmental marker, blue) upon transient expression in *Nicotiana benthamiana* leaves. Bar: 10 μm. (e) Analysis of CPT3 and Lew1 protein‐protein interaction using split yellow fluorescent protein (YFP) BiFC assay in tobacco leaf cells. Shown is the co‐localisation of fluorescence signals from the CPT1/Lew1 complex (green) and from the compartmental marker ER‐CFP (blue). Bar: 10 μm. See Section 2. (f) Analysis of CPT3 and Lew1 interaction using yeast two‐hybrid system. (g,h) Polyisoprenoid profiles and glycosylation status of CPY analysed for *rer2*Δ transformed with empty vector, *CPT3, LEW1* and *CPT3/LEW1*. Representative HPLC/UV chromatograms are shown. The positions of mature CPY (mCPY) and its hypoglycosylated glycoforms (lacking between one and four *N*‐linked glycans, ‐1 to ‐4) are indicated. See Section 2 [Color figure can be viewed at wileyonlinelibrary.com]

At the time, no T‐DNA insertion mutant in the *CPT3* gene was available from the NASC collection. For this reason, to analyse *in planta* the involvement of CPT3 in Dol formation, four independent RNAi lines targeting CPT3 for mRNA knockdown (RNAi‐1, ‐12, ‐14 and ‐23) and a transgenic line overexpressing CPT3 (OE‐7) were generated. The expression level of *CPT3* and the polyisoprenoid content were examined in 4‐week‐old leaves of these mutants. qRT‐PCR analyses revealed that the *CPT3* transcript is significantly reduced (by 40%–50%) in the four RNAi lines, and it is nearly 5‐fold elevated in the OE line, in comparison to wild‐type plants (Figure 2a). No visible phenotypic changes were observed between wild‐type plants and the studied mutant lines under standard growth conditions (data not shown). In contrast, HPLC/UV analysis of total polyisoprenoids revealed a significant decrease in dolichol (Dol‐15 to Dol‐17, dominating Dol‐16) accumulation in *CPT3* RNAi lines—to approximately 50% of the WT for three lines (RNAi‐1, ‐12 and ‐23) and to approximately 80% for RNAi‐14. Not surprisingly, *CPT3‐OE* plants accumulated significantly higher amounts of Dols, reaching 300% of the WT levels (Figure 2b). These results clearly suggest that CPT3 is involved in the biosynthesis of the major family of Dols in Arabidopsis. In line with this, we observed a positive correlation between the level of *CPT3* transcript and the content of Dol during plant development for three of the selected accessions (Figure 2c). This further supports the role of CPT3 in Dol formation; interestingly, no such correlation was noted for Prens (Figure 2c).

CPT3, similarly to numerous other eukaryotic CPTs engaged in Dol biosynthesis (Grabińska et al., 2016), is located in the endoplasmic reticulum (ER), as documented by confocal laser microscopy—in transiently transformed *N. benthamiana* leaves the fluorescence signal of CPT3‐GFP was detected in the ER‐like structures (Figure S6a) and fully overlapped with that of the ER marker ER‐CFP (Figure 2d). It is well‐established that some of the eukaryotic CPTs require an accessory protein for their enzymatic activity (Grabińska et al., 2016). Such CPT partners have been characterised for human and yeast CPTs—NgBR and Nus1, respectively. In silico analysis of Arabidopsis genome led to identification of Lew1 as NgBR/Nus1 homologue while phylogenetic analysis of CPTs revealed that CPT3 belongs to the subgroup of heterodimeric CPTs (Surowiecki et al., 2019). To verify this notion, the physical interaction of CPT3 with Lew1 was confirmed *in planta* using a BiFC assay (nEYFP‐C1/CPT3 was transiently co‐expressed with cEYFP‐N1/Lew1 in *N. benthamiana* leaves, Figures 2e and S6b) and Y2H system (Figure 2f).

Finally, functional complementation of the yeast mutant *rer2*Δ by Arabidopsis *CPT3* followed by an analysis of the polyisoprenoid profile of transformants (Figure 2g) revealed that solely co‐expression of *CPT3* and *LEW1* resulted in the synthesis of the major family of Dols (Dol‐14 to Dol‐16, Dol‐15 dominating, Figure 2g). Moreover, in line with the cellular function of Dol as an obligate cofactor of protein *N*‐glycosylation, only simultaneous expression of *CPT3* and *Lew1* fully rescued the defective glycosylation of the marker protein CPY in *rer2*Δ mutant cells (Figure 2h).

Taken together, the genetic and biochemical data presented here clearly show that Arabidopsis CPT3 is a functional ortholog of yRer2 and that CPT3 is responsible for Dol synthesis in Arabidopsis. It should be kept in mind however, that further experiments are needed to document that *CPT3* is causal of the natural variation between Col‐0 and Est. Additionally, despite significantly different content of Dol, the general performance of the *CPT3*‐deficient and *CPT3‐OE* Arabidopsis lines, at least upon standard growth conditions, does not differ from that of the WT plants. This observation, on the one hand, might indicate that Dol level in the tissues of *CPT3* RNAi lines is sufficient to support Dol‐dependent cellular processes. On the other hand eukaryotic cells certainly possess mechanisms to cope with the increased content of Dol associated with aging.

### 3.7 Genetic analyses of the variations in metabolite levels in natural accessions: GWAS

As a following step, we used a multi‐trait mixed model (Korte et al., 2012) to calculate the genetic correlations between the different traits studied (see Table S4). Here, we found a strong correlation for the four traits—Prens, phytosterols, plastoquinone and Dols, which argues for a common genetic correlation of these four traits, and at the same time it shows that they have a negative genetic correlation with the remaining three traits, namely tocopherols, chlorophylls and carotenoids.

**Figure 3:**
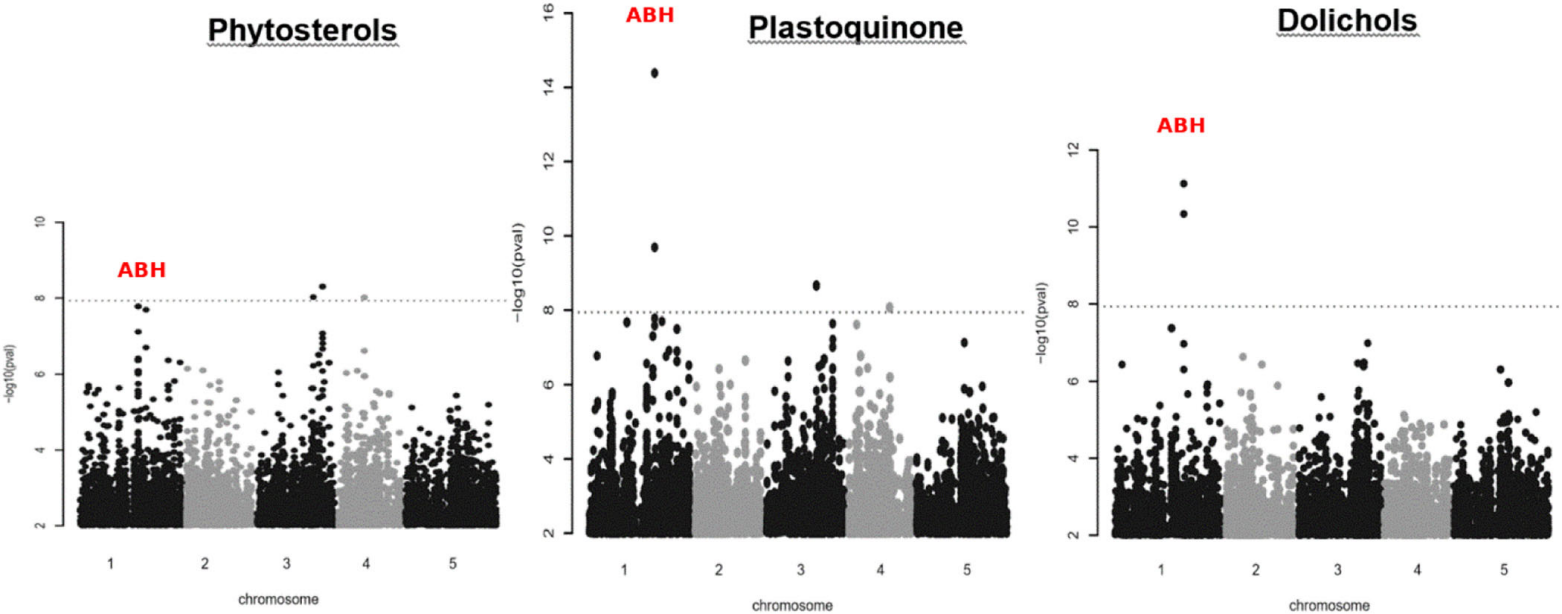
Manhattan plot of genome‐wide associations for phytosterols, plastoquinone and dolichols. The dotted horizontal lines indicate a significance level of 0.05 after Bonferroni correction for multiple testing (see Section 2). Manhattan plot of genome‐wide association results for polyprenols, chlorophylls and tocopherols are shown in Figure S7 [Color figure can be viewed at wileyonlinelibrary.com]

Next, we used the mean phenotypic values of the 116 natural Arabidopsis accessions per trait to perform GWAS. We used an imputed SNP data set that contains ~2 million polymorphisms. At a 5% Bonferroni corrected significance threshold significant associations have been found only for three of the seven different compounds analysed (Dols, plastoquinone and phytosterols), while no significant associations have been found for the other four compounds (chlorophylls, carotenoids, Prens and tocopherols). Noteworthy, our GWAS has a rather low power because of the small number of accessions included. Power analysis indicate that only markers that explain at least 10% of the overall trait variance can be reliable detected in our GWAS setting. In summary, 2, 7 and 5 distinct genetic regions were significantly associated with Dols, plastoquinone and phytosterols, respectively. One region on chromosome 1 is found for all three traits. The respective Manhattan plots are shown in Figures 3 and S7 show the Manhattan plots of the remaining traits.

**Figure 4:**
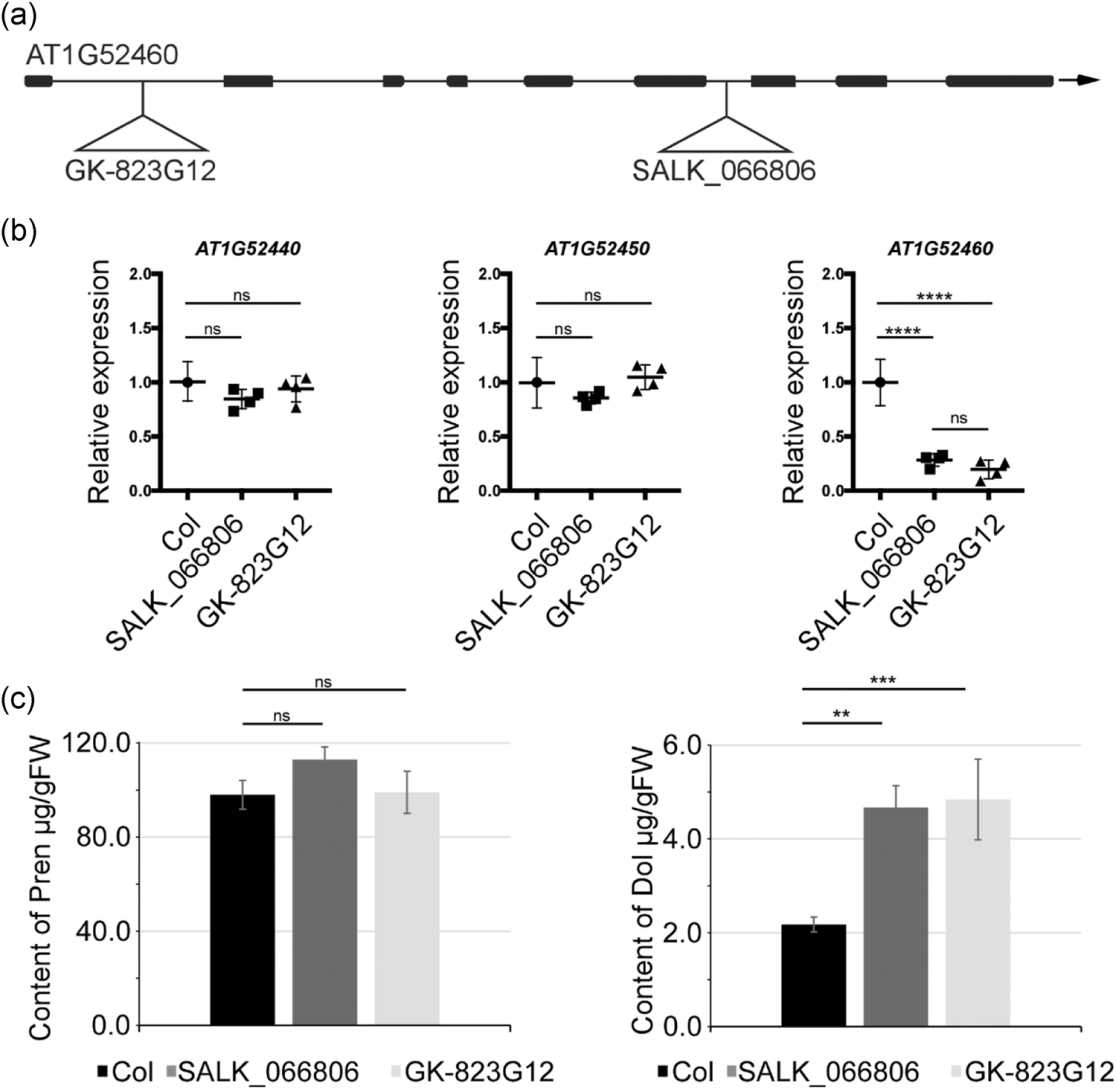
Effect of AT1G52460 deficiency on the level of transcripts of neighbouring genes and the content of polyisoprenoids. (a) AT1G52460 gene structure. Exons and introns are indicated by thick and thin lines, respectively. The T‐DNA insertion sites in two independent mutant lines: SALK_066806 and GK_823G12 are depicted. (b) Levels of AT1G52440, AT1G52450 and AT1G52460 transcripts (qPCR analysis) in the leaves of 3‐week‐old plants—WT (Col) and AT1G52460‐deficient plants were compared. *p* ≤ 0.0001 (one‐way ANOVA followed by Tukey post‐tests); ns, non‐significant. (c) The content of Dols and Prens estimated in leaves of 3‐week‐old plants using HPLC/UV, shown are means ± SD of five independent biological replicates. The phenotypic appearance of 4‐week‐old detached leaves of AT1G52460‐deficient line (SALK_066806, *abh* heterozygous mutant) and wild‐type (Col‐0) plants grown in soil is shown in Figure S8. Seed germination and segregation analysis of F1 progeny of self‐pollinated of heterozygous SALK_066806 and GK_823G12 lines is shown in Table S5

The region that is detected for all three traits contains two SNPs that were associated with Dol content. The first of the associated polymorphisms is at position 19 545 459 on chromosome 1 and it codes for a non‐synonymous AA‐exchange (Q270K) in the first exon of AT1G52450, a gene involved in ubiquitin‐dependent catabolic processes. This polymorphism is significant for all three traits. The second polymorphism is located at position 19 540 865: it is upstream of AT1G52450 and in the 3′ UTR of the neighbouring gene AT1G52440, which encodes a putative ABH. A second putative ABH (AT1G52460) is also within 10 kb of these associations. The remaining significant associations for this three traits are not replicated across traits and putative candidates are shown in Table S7. The identification of AT1G52450 and two neighbouring genes as putative effectors of the accumulation of Dols, plastoquinone and phytosterols prompted us to analyse the phenotypes of the respective Arabidopsis T‐DNA insertion mutants (Figure 4). Interestingly, a significant increase in the content of Dols (approximately 2‐fold, comparing to control WT plants) was noted for two analysed heterozygous AT1G52460‐deficient lines: SALK_066806 and GK_823G12. Moreover, in the SALK_066806 line, phytosterol content was also increased (167.8 ± 20.3 vs. 117.4 ± 23.2 μg/g of fresh weight) and plastoquinone content was considerably decreased (27.3 ± 2.0 vs 56.7 ± 5.2 μg/g of fresh weight). It is worth noting that mutations in the AT1G52460 gene did not affect the content of Prens—this gene has not come up as that putatively affecting Pren accumulation (Figure 4). Additionally, these mutant plants developed deformed, curled leaves (Figure S8). Expression analysis of genes of interest in the genetic backgrounds of heterozygous AT1G52460‐deficient lines (both SALK_066806 and GK_823G12) revealed that in comparison to WT (Columbia‐0) plants, the level of AT1G52460 mRNA was considerably decreased while that of AT1G52440 and AT1G52450 remained unchanged (Figure 4b).

To establish the reason for the inability to obtain homozygous AT1G52460‐deficient mutant plants, we analysed and genotyped the progenies of heterozygous plants originating from the SALK_066806 (*n* = 61) and GK_823G12 (*n* = 151) lines. The lack of AT1G52460‐deficient homozygotes among analysed plants of each mutant line suggested that disruption of this gene was lethal (Table S5). Since the fraction of aborted seeds per silique was higher for both mutants (approximately 17.9% and 25.5% for GK_823G12 and SALK_066806, respectively) than for WT line (2.6%), the seeds produced by mutants showed a reduced germination rate comparing to WT plants (Table S5). It suggests that homozygous mutation in AT1G52460 most probably results in embryolethality. Other analysed homozygotic mutants (carrying insertions in the genes AT1G52440 and AT1G52450) did not show significant differences neither in isoprenoid content nor in macroscopical appearance (data not shown).

Taken together, identification of the involvement of putative ABH, encoded by AT1G52460, in Dol biosynthesis sheds new light on metabolic pathway in eukaryotes, although the cellular mechanism underlying this process as well as the causative role of ABH variants in the natural variation of Dol accumulation awaits clarifications.

### 3.8 Correlation analyses of isoprenoid accumulation in the various accessions and in the mapping population: A statistical meta‐analysis

As a final step, we conducted a detailed statistical meta‐analysis of the studied traits in the different Arabidopsis accessions and in the lines of the EstC mapping population. Numerous correlations were found for the content of seven isoprenoid compounds estimated in the seedlings of natural accessions and the mapping population (Figure 5a,b, respectively). Moreover, we clearly identified some outliers (Grubbs test at significance level α = 0.001) (Grubbs, 1950). For plastoquinone, seven values corresponding to three accessions (Er‐0, Est‐1 and Fei‐0) were unequivocally assigned as outliers, for carotenoids—three values corresponding to a single accession (Ren‐1), for phytosterols a single outlier was identified in the natural accessions and for Dols in the mapping population (Figure S9). All these outliers, denoted by red triangles in Figure 5, were filtered out in the statistical analysis of metabolite distribution and the correlation analyses (Figure 5a,b). For both datasets, the analysis of metabolite correlations revealed the highest correlation for chlorophylls versus carotenoids (*R* > 0.97), while four other metabolites—phytosterols, Prens, plastoquinone and Dols—also correlated with each other significantly (*p* < 0.0001) Table S6. Tocopherol accumulation correlated only occasionally with the other metabolites (Table S6). Based on the structural similarity between Prens and Dols, some level of similarity between the mechanisms of their accumulation might be expected. However, the obtained values for the correlation between Prens and Dols among the tested accessions (0.325, *p* = 0.0001) and among the AI‐RILs (0.608, *p* = 0.0001) suggest differences between these two subgroups of polyisoprenoids. Relationships between levels of metabolites analysed in this report were also confirmed using hierarchical clustering Figure S10.

Figure 5Correlations between the content of seven metabolites estimated in the seedlings of Arabidopsis accessions (a) and the EstC mapping population (b). The original distributions (green bars), together with the approximation of the normal distribution of the data (blue curve) with outliers removed, are presented on the diagonal. Correlation patterns for each metabolite pair are presented at the appropriate intersection; note that outliers (red dots) were not taken into consideration for the analysis. Above each diagonal panel, the Shapiro–Wilk statistics (W, p) for normal distribution is presented, while for out‐of‐diagonal panels Pearson (P) and Spearman (S) correlation coefficients together with the associated significance levels are shown (please note that ‘0’ means *p* < 1e−7). Bearing in mind the statistically significant deviations from normal distribution shown in the diagonal panels, the significance of the observed correlations should be interpreted in terms of the Spearman rather than Pearson coefficient (see Section 2). Cumulative distribution functions of the content of seven studied metabolites analysed in the seedlings of Arabidopsis accessions and AI‐RILs are shown in Figure S9 [Color figure can be viewed at wileyonlinelibrary.com]

**Figure:**
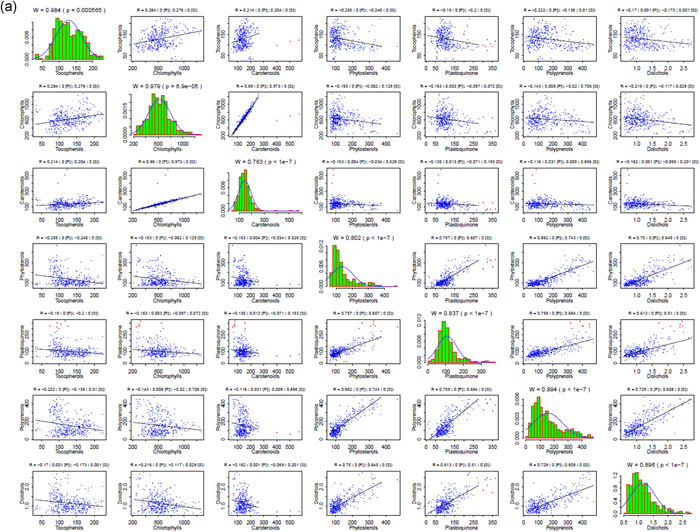

**Figure:**
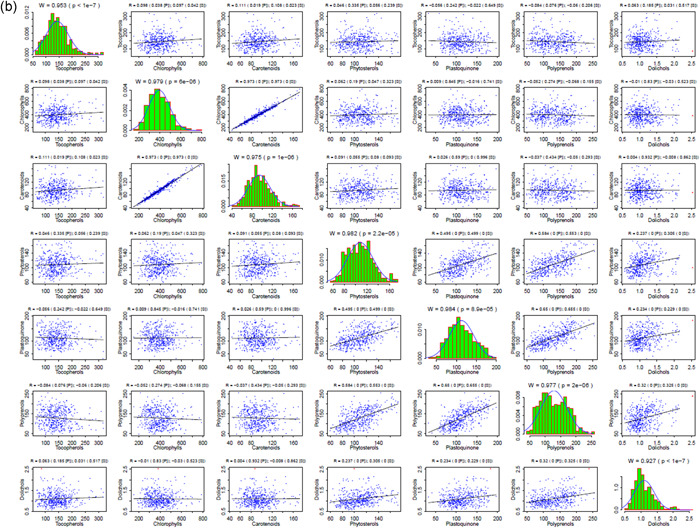

Importantly, all the strongest genetic correlations detected for particular metabolites (Table S4) were also identified as the most significant (*p* < 0.0001) for metabolic data‐based analysis and this is valid both for the natural accessions and for the EstC mapping population lines (Table S6). Moreover, a consistent trend of correlations (either positive or negative) between individual metabolites in the natural accessions was observed for both genetic‐ and metabolic‐based analysis (Tables S4 and S6). Taken together, results of the meta‐analysis indicate genetic coregulation of the biosynthesis of specific isoprenoids.

## 4 DISCUSSION

**Figure 6:**
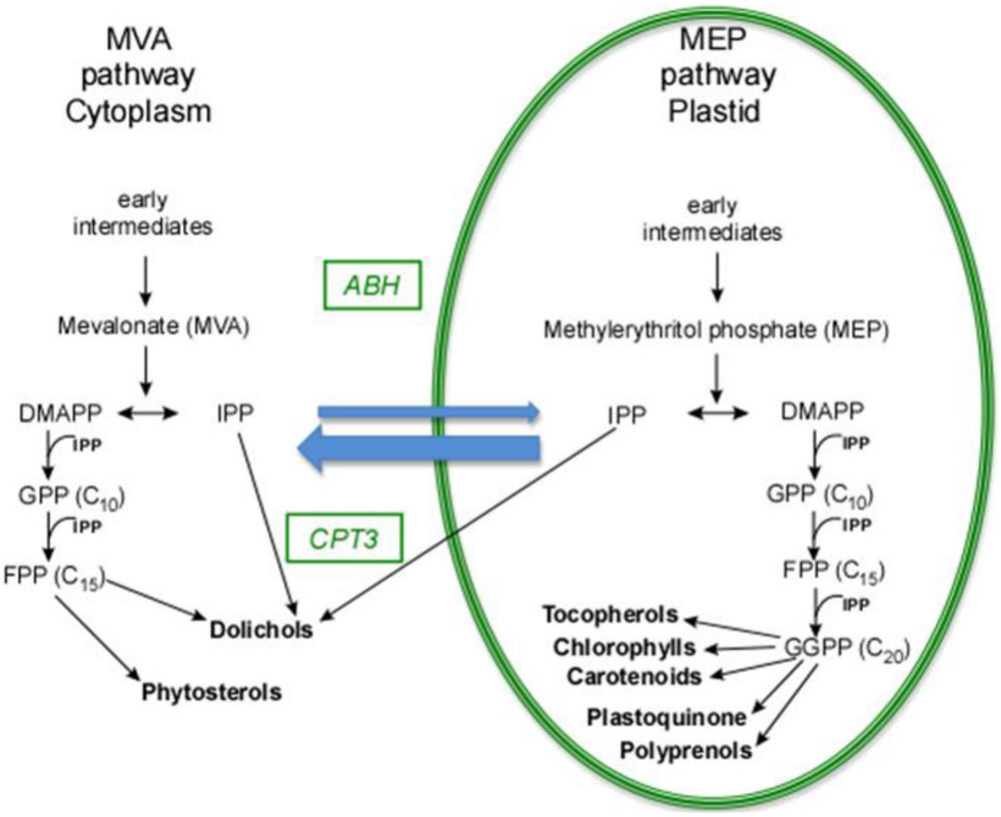
Biosynthetic routes leading to isoprenoids in Arabidopsis cell; the involvement of the genes*cis*‐prenyltransferase 3 and putative role of α/β‐hydrolase is indicated. Depicted are seven metabolites analysed in this study. Two pathways, the mevalonate (MVA) and methylerythritol phosphate (MEP) pathways, are generating IPP in parallel, both contributing to particular isoprenoids (Akhtar et al., 2017; Hemmerlin et al., 2012; Jozwiak et al., 2017). Blue arrows illustrate the exchange of intermediates between the MVA and MEP pathways. DMAPP, dimethylallyl diphosphate; FPP, farnesyl diphosphate; GPP, geranyl diphosphate; GGPP, geranylgeranyl diphosphate; IPP, isopentenyl diphosphate [Color figure can be viewed at wileyonlinelibrary.com]

Dolichol is a vital component of eukaryotic cell synthesis and accumulation of which is tightly regulated in response to physiological requirements and environmental stimuli. The identification of a long searched for CPT3 makes the biosynthetic route of Dol in plants complete and implicates possible integration of this pathway into Dol‐producing biotechnological platforms. Association of a putative ABH, encoded by AT1G52460, with Dol accumulation in Arabidopsis provides novel insight into the possible determinants of Dol level in all eukaryotes (Figure 6). Understanding the cellular mechanisms underlying this association requires elucidation, but without the genetic association approach used in this study, it would be difficult to reveal them.

Here, we detected QTLs for four different compounds: Prens, Dols, chlorophylls and carotenoids, while we found significant GWAS associations for three: phytosterols, plastoquinone and Dols. Consequently, Dols are the only compounds where both approaches detected associations. Still, the reported QTL on chromosome 2 does not overlap with the GWAS results, which are located on chromosomes 1 and 3, respectively (summarised in Table S7). While, at a first glimpse, this lack of accordance might be disturbing, there could be many good reasons for it. It is well known that both methods have different power to detect associations (see fig. 4 in Weigel & Nordborg, 2015). For example, on chromosome 1, we identified a significant GWAS association for three different compounds, but we detected no corresponding QTL in the mapping population even though the associated polymorphism segregates in the AI‐RIL population. The three traits for which this association is detected (the content of phytosterols, plastoquinone and Dols) show a strong genetic correlation, so one would expect to find shared genetic factors that regulate all three traits, despite a slightly lesser phenotypic correlation of the traits. The associated sequence variant is located in the gene AT1G52450, which is thus an excellent candidate to modulate all three traits and would not have been found using QTL mapping alone. AT1G52450 is annotated to encode a ubiquitin carboxyl‐terminal hydrolase (UCH)‐related protein, while the neighbouring gene AT1G52460 encodes an ABH (PubMed Gene database). Neither of these proteins has been characterised yet.

Eukaryotic cells usually possess a family of UCHs (e.g., three in Arabidopsis) (Isono & Nagel, 2014) responsible for releasing ubiquitin (Ub) from ubiquitinated proteins. A tight balance between ubiquitination and deubiquitination is required for cellular survival since ubiquitin controls numerous bioactivities, such as protein degradation by the 26S proteasome, cell cycle regulation, signal transduction or membrane trafficking. In turn, the ABH superfamily proteins are found across all domains of life. They are implicated in primary and secondary metabolism by serving highly diverse enzymatic activities, for example, as esterases, thioesterases, lipases, proteases. Additionally, proteins with the ABH fold function as receptors in the strigolactone, gibberellin and karrikin‐smoke response pathways (Mindrebo et al., 2016 and references therein). In Arabidopsis, more than 600 proteins with ABH folds have been predicted by the InterPro database (Mitchell et al., 2019) with the majority remaining uncharacterized.

Taken together, hydrolytic enzymes, as ABH, encoded by AT1G52460, and/or UCH, encoded by AT1G52450, might control isoprenoid biosynthesis in eukaryotic cells. Interestingly, both *ABH* and *UCH* show a high dN/dS ratio (ratio of nonsynonymous to synonymous divergence) in the Arabidopsis population, arguing for strong selection on these genes (see Table S8). Further studies are needed to identify the cellular target(s) of AT1G52460 and the mechanisms underlying its involvement in the metabolism of Dol, phytosterol and plastoquinone.

It is worth noting that in previous reports, the AT1G52460 gene was identified as one of the maternally expressed imprinted genes (MEGs) that was shown to be predominantly expressed from maternal alleles in reciprocal crosses (Wolff et al., 2011). Notably, the AT1G52460 was among the MEGs (∼30% of all the MEGs tested in that study) for which authors reported a *dN*/*dS* value greater than one (Wolff et al., 2011). The dN/dS value can be used to measure the rate of molecular evolution of genes (Warren et al., 2010); therefore, the results of Wolff et al. (2011) provide particularly strong evidence for the fast evolution of AT1G52460. Taking into account that, we detected only heterozygotic lines for the AT1G52460 gene, we consider that a loss‐of‐function allele may lead to a lethal phenotype. A 2:1 ratio (the frequency of heterozygous:WT plants in F_2_) fitted the data (χ² = 2.6 and χ² = 0.2 for GK_823G12 and SALK_066806 lines, respectively, at the value of *p* > 0.05). This finding could be particularly important, and it deserves further investigation since very few imprinted genes have been confirmed in plants and even fewer of them have been functionally investigated (He et al., 2017).

The most promising gene identified in the QTL analysis, AT2G17570 (*CPT3*), is a long‐searched enzyme responsible for backbone synthesis for the major family of Dols in Arabidopsis, with Dol‐15 and Dol‐16 dominating. Interestingly, the different product specificity of the Arabidopsis enzymes CPT3, CPT6 (which produces *in planta* a single Dol‐7 [Surmacz et al., 2014]) and the recently characterised CPT1 (producing a family of Dols with Dol‐21 dominating [Surowiecki et al., 2019]) suggests that the particular AtCPTs play dedicated, non‐redundant roles in isoprenoid synthesis in Arabidopsis tissues. For further comments regarding CPT3 see also Table S8.

Even though no overlapping associations have been found for the GWAS and QTL results, one can try, using the GWAS results, to prioritise candidate genes in the QTL interval. In the confidence interval of the detected QTL for Dol on chromosome 2, we could analyse 6668 independent segregating polymorphisms with a minor allele frequency greater than 5%. None of these reached the genome‐wide significance threshold; the most significant polymorphism had a *p*‐value of 4.88 × 10^–6^ and was located in the proximity of AT2G17570, which encodes CPT3. Although this score is marginal, it is locally significant, if we restrict our analysis to sequence variants within the QTL region. So, the combined results of GWAS and QTL strongly indicate that *CPT3* is the gene underlying the detected QTL for Dol, despite the plethora of other tempting candidate genes. Detailed SNP analyses of *CPT3* revealed that this gene shows a high amount of variation with a total number of 30 non‐synonymous substitutions and 5 alternative starts and 1 premature stop codon in the Arabidopsis population (Table S8).

Overall, this study identified several candidate genes for potential novel factors that may affect polyisoprenoid accumulation. Regulation of the isoprenoid pathways is complex, but by using a combination of GWAS and QTL, it is possible to prioritise the underlying genes. The genetic and biochemical evidence described in this report documents the role of CPT3 and ABH in Dol pathway (Figure 6), however, more research is needed to prove their causal role in the natural variation of this trait. It is worth underlying that both genetic‐ and metabolic‐based analysis revealed correlations of the analysed traits indicating genetic co‐regulation of the biosynthesis of specific isoprenoids. Last but least, it should be kept in mind that this study is based on terpene levels at the seedling stage and might not be representative for later growth stages. Anyhow, obtained results clearly suggest the role of CPT3 and ABH in Dol accumulation.

Understanding the mechanisms of Dol synthesis/accumulation in eukaryotes is important because a deficiency of dolichol/DolP causes severe defects in all organisms studied, most likely due to defective protein glycosylation. In plants, it is lethal due to male sterility (Jozwiak et al., 2015; Lindner et al., 2015), while in humans, mutations in the genes encoding enzymes involved in Dol/DolP synthesis lead to rare genetic disorders collectively called Congenital Disorders of Glycosylation (CDG type I). It has been proposed to supplement the diet with plant tissues that can be used as a source of dolichol/DolP (summarised in Buczkowska et al., 2015). The identification of genes involved in the synthesis/accumulation of Dols—such as the *CPT3* and *ABH* detected here—opens up the prospect of manipulating the Dol content in plants and consequently makes it possible to think about constructing plants with an increased Dol content. Moreover, the involvement of ABH in the synthesis of Dol in Arabidopsis may also suggest an analogous role for ABH in mammalian cells, pointing to a new potential therapeutic strategy for CDG patients.

## CONFLICT OF INTERESTS

The authors declare that there are no conflict of interests.

## Supporting information

Supporting information.

## Data Availability

All data obtained and/or analysed in this study are available from the corresponding authors upon reasonable request. The phenotypic data used for GWAS are available at the AraPheno, https://arapheno.1001genomes.org/. GWAS script: the R scripts used are available at https://github.com/arthurkorte/GWAS. The genotype data used for GWAS are available via the AraGWAS Catalogue, https://aragwas.1001genomes.org/#/download-center.
