# Supplementary material for: cis‐prenyltransferase 3 and α/β‐hydrolase are new determinants of dolichol accumulation in Arabidopsis
Source: Plant Cell Environ. 2021 Nov 29;45(2):479–95. doi: 10.1111/pce.14223 (PMC9300173; doi:10.1111/pce.14223)
Supplement: Supplementary file 1 — Supporting information. [file PCE-45-479-s001.docx]

**SUPPORTING INFORMATION**

**Gawarecka et al.**

***cis*-Prenyltransferase 3 and α/β-hydrolase are new determinants of dolichol accumulation in Arabidopsis**

**Table of contents**

**Figure S1.** A simplified scheme depicting main steps leading to formation of Prens, Dols and other isoprenoid compounds analyzed in this report.

**Figure S2.** Profiles of lipids isolated from Arabidopsis Col-0 seedlings. (A) Polyprenols (Pren) and dolichols; (B) Phytosterols, (C) Plastoquinone, (D) Tocopherols.

**Figure S3.** Content of selected isoprenoids in the seedlings of Arabidopsis accessions: (A) chlorophylls, (B) carotenoids, (C) phytosterols, (D) plastoquinone and (E) tocopherols.

**Figure S4.** Frequency distribution of the content of (A) chlorophylls, (B) carotenoids, (C) phytosterols, (D) plastoquinone and (E) tocopherols in the seedlings of AI-RILs and their parental lines, Col-0 and Est-1.

**Figure S5.** LOD profiles for QTLs underlying the accumulation of selected isoprenoids in the AI-RILs: (A) polyprenols, (B) dolichols, (C) chlorophylls, and (D) carotenoids.

**Figure S6.** Subcellular localization of CPT3 in confocal microscopy (A) and analysis of CPT3 and Lew1 protein–protein interaction - negative controls of BiFC assay (B).

**Figure S7.** Manhattan plot of genome-wide association results for polyprenols, chlorophylls and tocopherols.

**Figure S8.** The phenotypic appearance of 4-week-old detached leaves of AT1G52460-deficient line.

**Figure S9.** Cumulative distributions (CDF) of the content of seven studied metabolites analyzed in the seedlings of Arabidopsis accessions (E) and AI-RILs (M).

**Figure S10.** Dendrograms and corresponding heatmaps calculated for the accessions (A) and the mapping population (B).

**Table S1.** Characteristics of the detected QTLs underlying polyprenol (Pren), dolichol (Dol), chlorophyll and carotenoid accumulation in the AI-RIL population.

**Table S2**. Selection of candidate genes from chosen QTL intervals.

**Table S3.** Candidate genes potentially involved in dolichol accumulation, selected from the mapped QTL interval DOL1.

**Table S4.** Genetic correlations between metabolite levels.

**Table S5.** Segregation of *at1g52460* alleles and seed germination rates of AT1G52460-deficient plants.

**Table S6.** Metabolic data-based correlations between metabolite levels.

**Table S7.** Summary of candidate genes involved in accumulation of Dol, plastoquinone, phytosterols and Pren – comparison of QTL and GWAS approaches.

**Table S8.** Detailed SNP analysis of AT2G17570 (*CPT3*), AT1G52450 (*UCH*) and AT1G52460 (*ABH*) sequences in the Arabidopsis population.

**Table S9.** *Arabidopsis thaliana* accessions used in this study.

**Table S10**. Primers used for the construction of the *AtCPT3* silencing vector, genotyping of AT1G52460 T-DNA insertion mutants and expression studies.

**Supplementary Methods**. Quantitative analysis of isoprenoids.


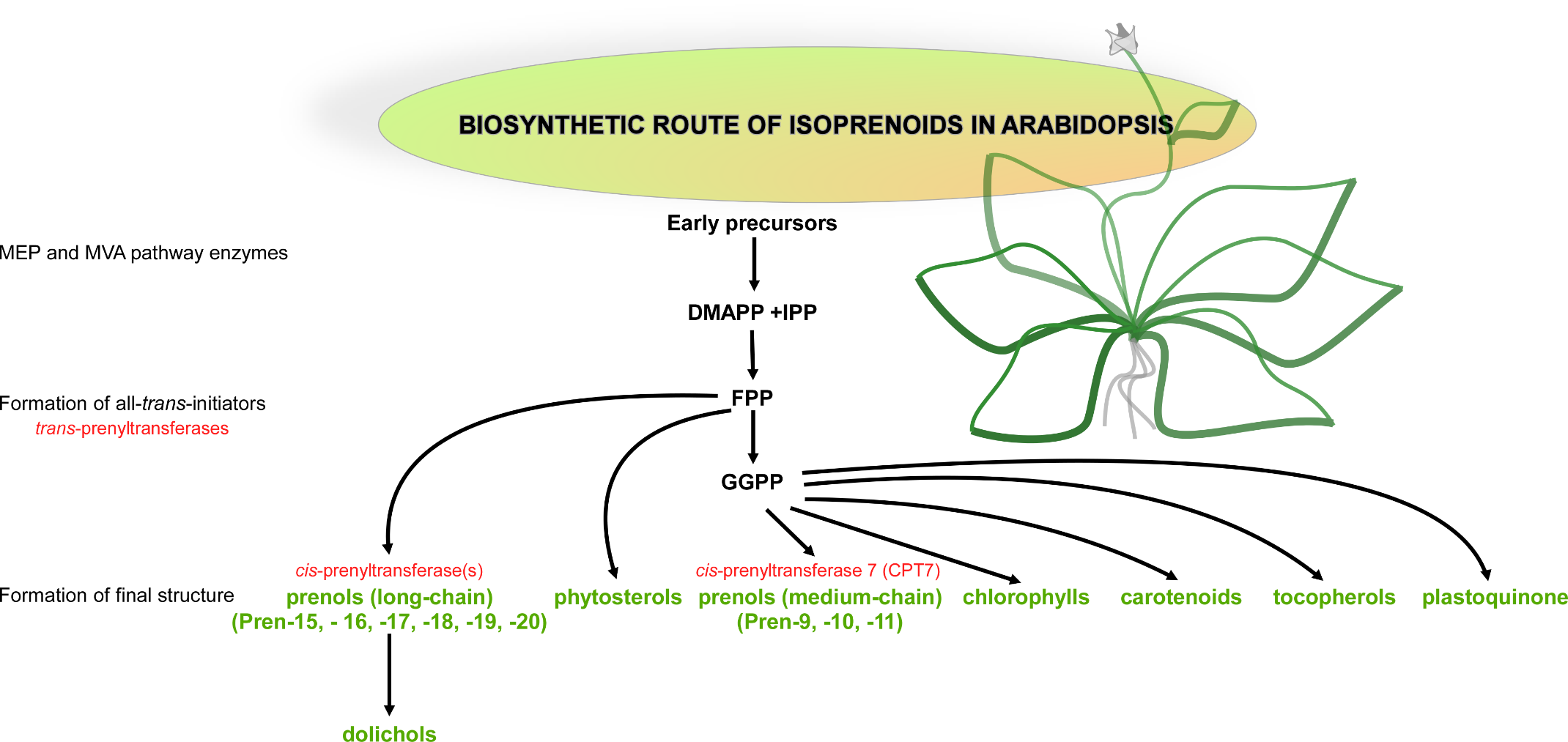


**Figure S1. A simplified scheme depicting main steps leading to formation of Prens, Dols and other isoprenoid compounds analyzed in this report.** Metabolic precursors of isoprenoids, DMAPP and IPP (dimethylallyl and isopentenyl diphosphate, respectively) derived from the mevalonate (MVA) and methylerythritol phosphate (MEP) pathways are condensed to form all-*trans* initiators - FPP and GGPP (farnesyl and geranylgeranyl diphosphate, respectively). Role of *cis*-prenyltransferases involved in formation of polyisoprenoids is depicted. Please note, that CPT7 catalyzes formation of exclusively Pren-9, -11 (Akhtar et al., 2017), see Introduction for further details.

**(A)**

**
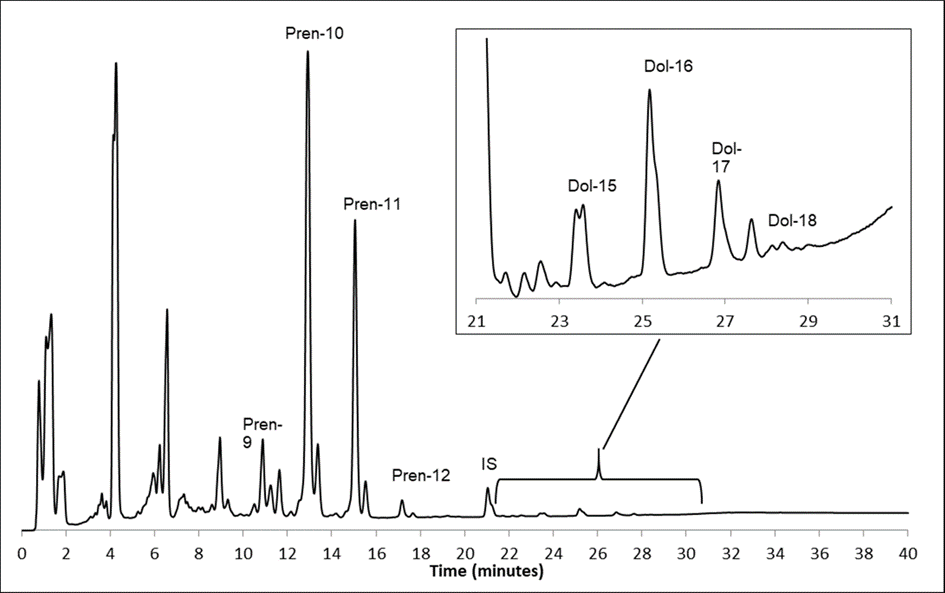
**

**(B)**


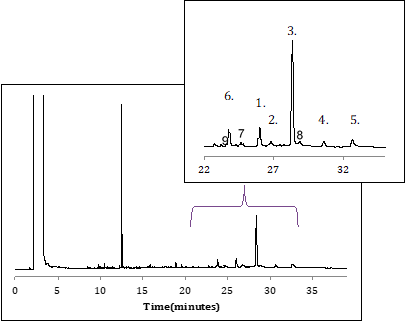


**(C)**


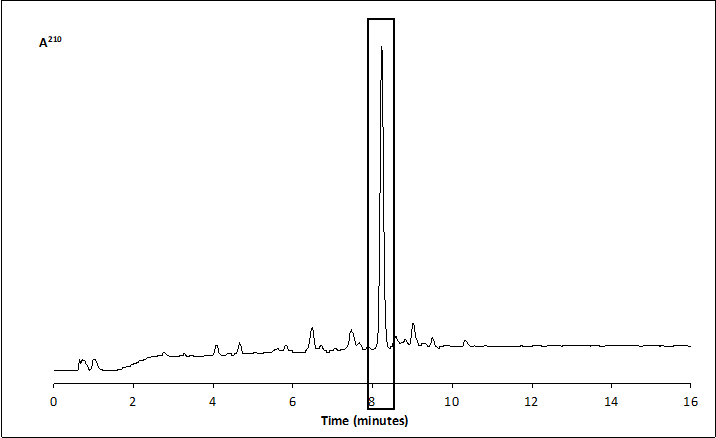


**(D)**


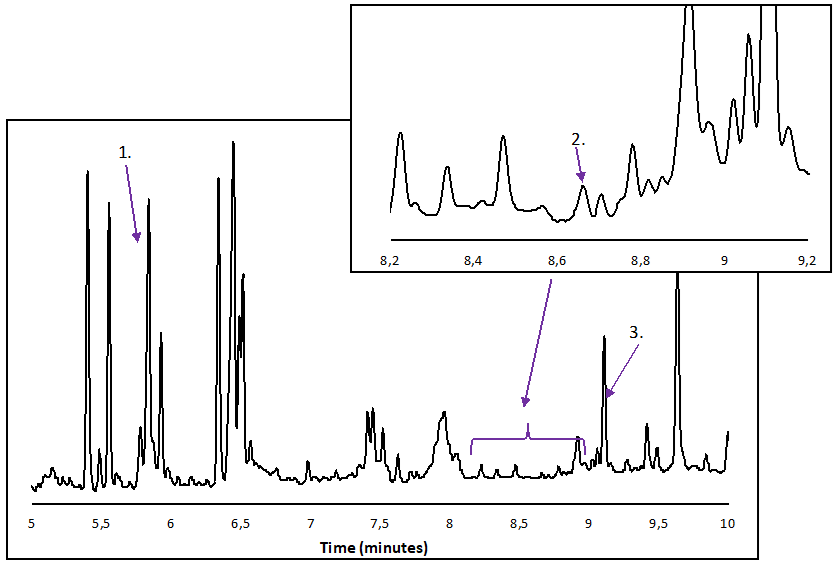


**Figure S2. Profiles of lipids isolated from Arabidopsis Col-0 seedlings.** The same profile of lipids of each particular type was observed for all analyzed accessions.

**(A) *Polyprenols (Pren) and dolichols (Dol, inset*) – HPLC/UV.** Signals corresponding to Pren-9 to -12 and Dol-15 to -18 were integrated to calculate the total amount of Prens and Dols, respectively. IS indicates the signal of the internal standard (Pren-14) (see Materials and methods).

**(B) *Phytosterols* - GC/FID.** 1. campesterol; 2. stigmasterol; 3. β-sitosterol; 4. stigmast-4,22-dien-3one; 5. stigmast-4en-3-one; 6. cholestanol – internal standard; 7. brassicasterol; 8. β-sitostanol 9. cholesterol. The same profile of phytosterols was recorded for all analyzed accessions. Inset presents the magnified region of chromatogram. Indicated signals (1-5 and 7-9) were integrated to calculate the amount of phytosterols.

**(C) *Plastoquinone* - HPLC/UV.** Indicated signal was integrated to calculate the amount of PQ.

**(D) *Tocopherols* - GC/FID.** 1. γ - tocopherol; 2. δ - tocopherol (inset); 3. α - tocopherol of Arabidopsis Col-0 seedlings. Indicated signals (1-3) were integrated to calculate the amount of lipids.

**(A)**


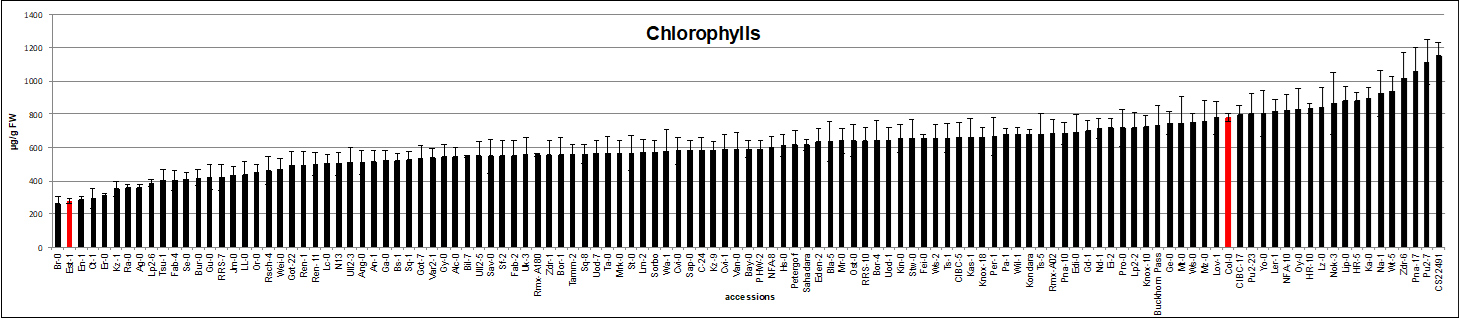


**(B)**


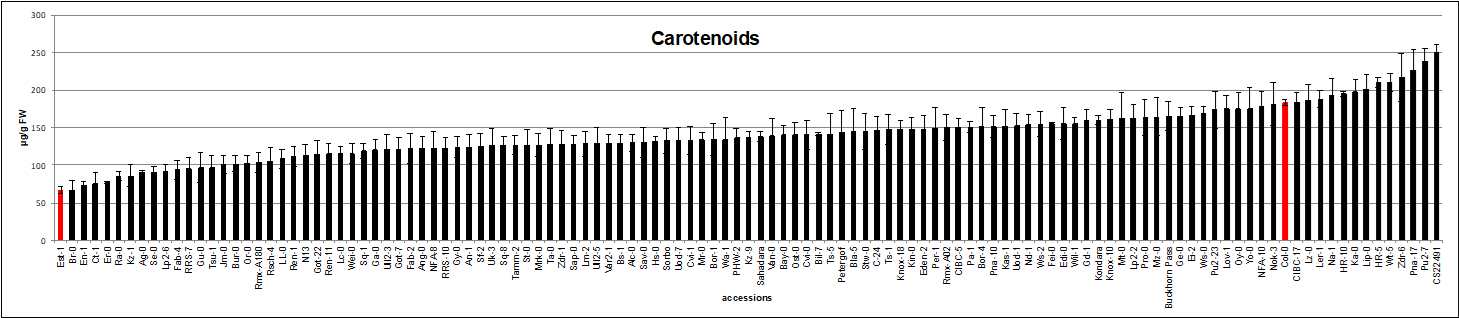


**(C)**


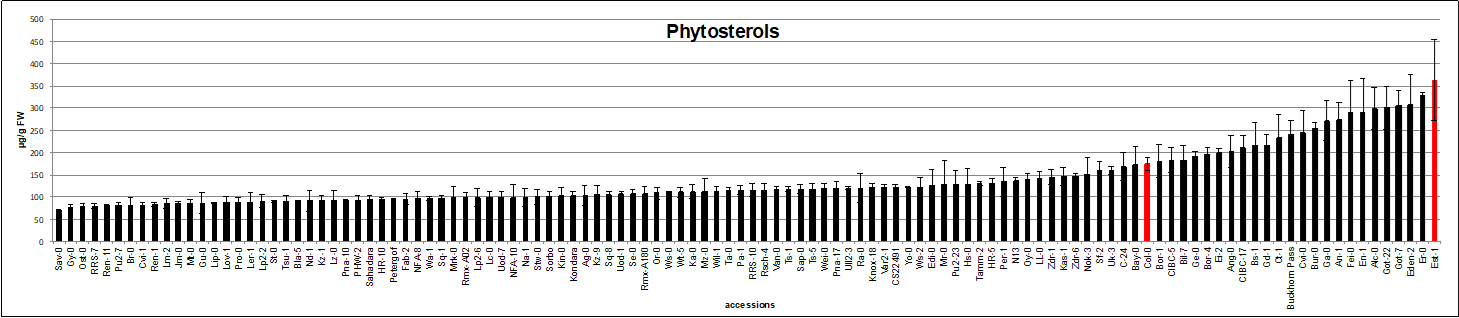


**(D)**

**
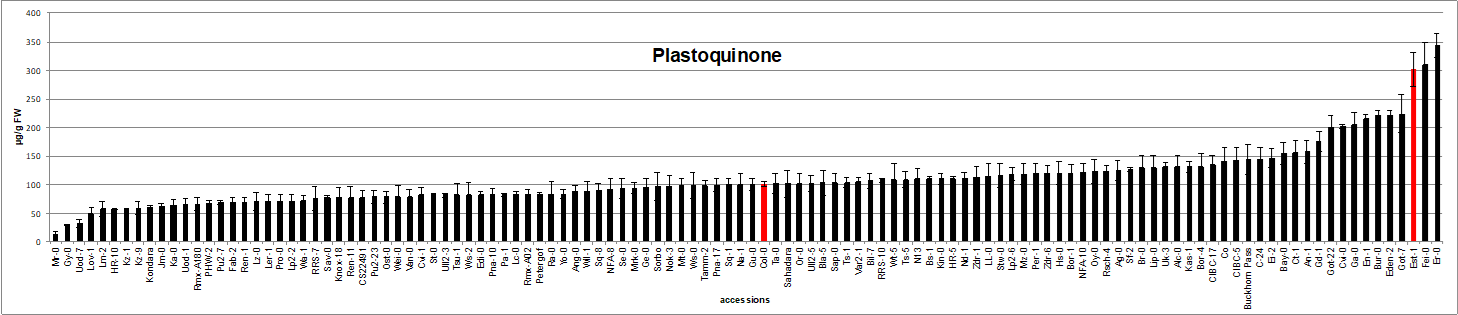
**

**(E)**


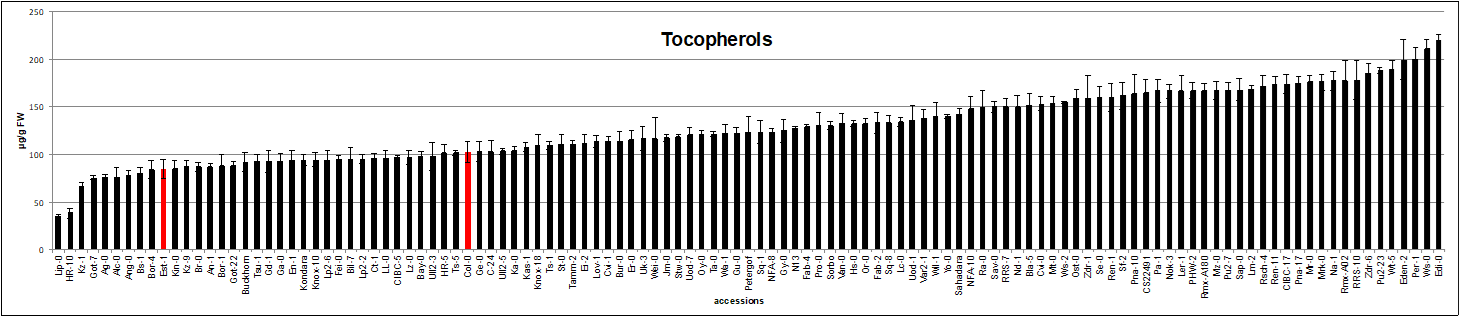


**Figure S3. Content of selected isoprenoids in the seedlings of Arabidopsis accessions: (A) chlorophylls, (B) carotenoids, (C) phytosterols, (D) plastoquinone and (E) tocopherols.** Bars presenting the content of particular isoprenoids in Col-0 and Est-1 are marked in red. All experiments were performed in triplicate, shown is mean ± SD.

**
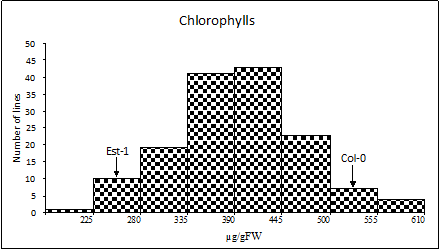

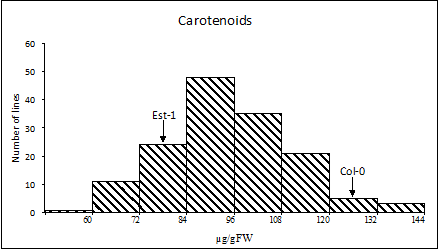
**

**(D)**

**(C)**

**(B)**

**(A)**

**
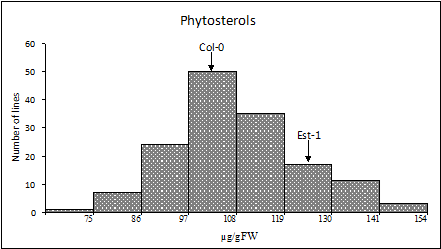

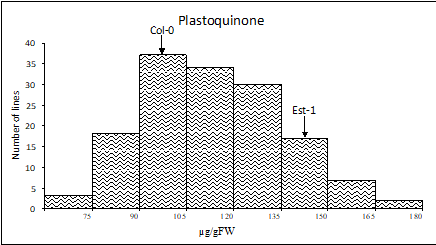
**

**(E)**

**
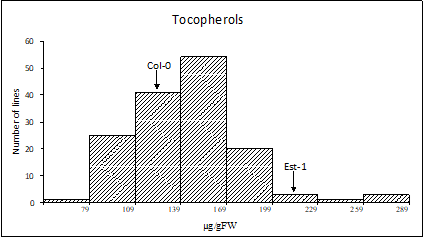
**

**Figure S4.** **Frequency distribution of the content of (A) chlorophylls, (B) carotenoids, (C) phytosterols, (D) plastoquinone and (E) tocopherols in the seedlings of AI-RILs and their parental lines, Col-0 and Est-1.** Each bar covers the indicated range of the content of a particular isoprenoid compound.

+
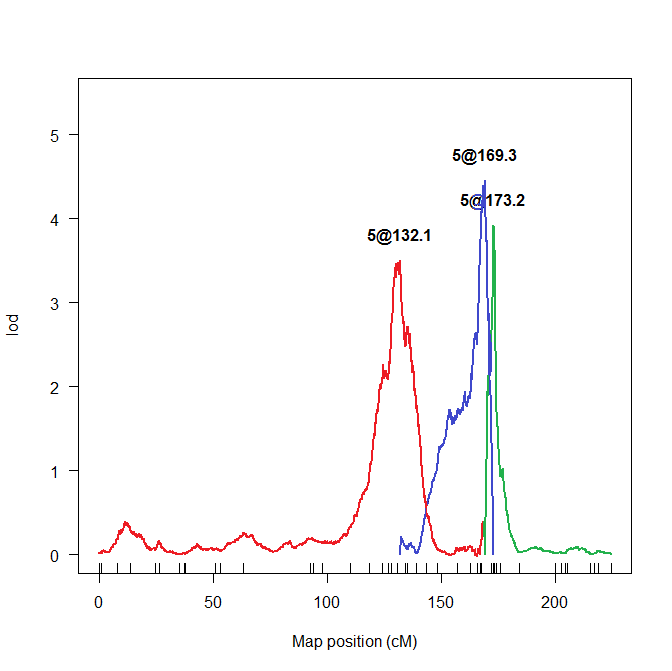

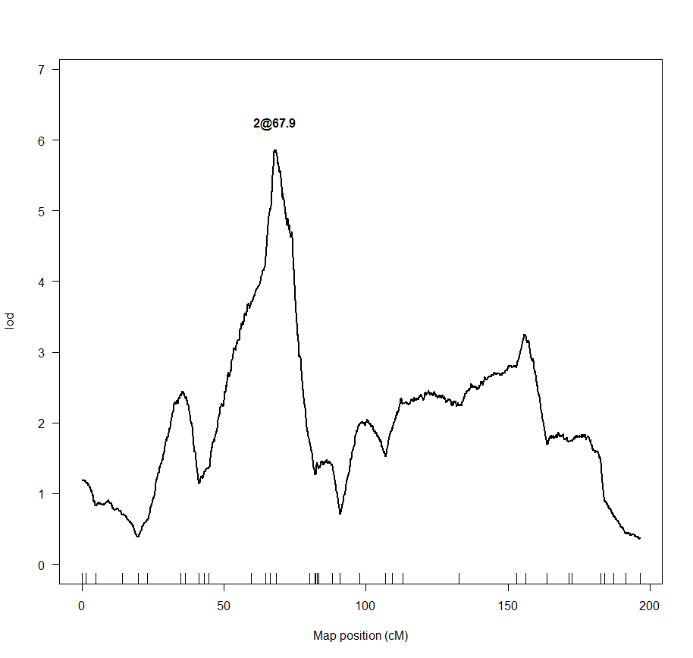


**(B)**

**(A)**

**LOD**

**Chr. 5**

**Chr. 2**

**(D)**

**(C)**


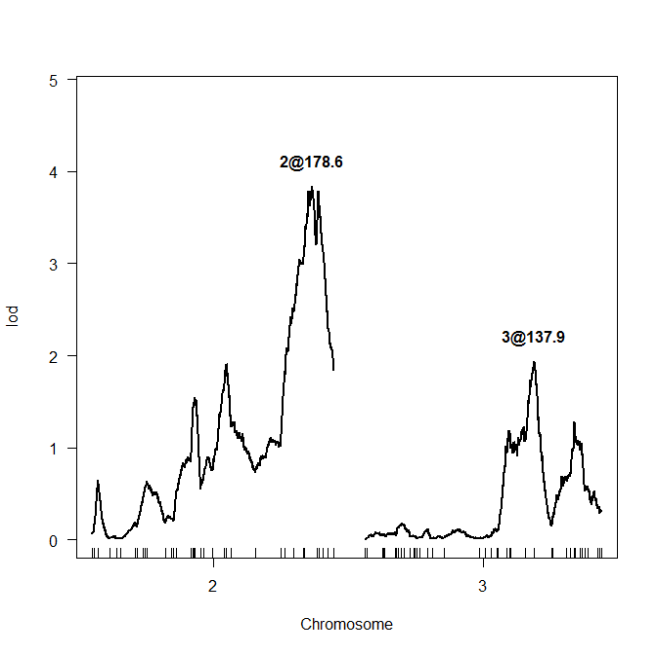

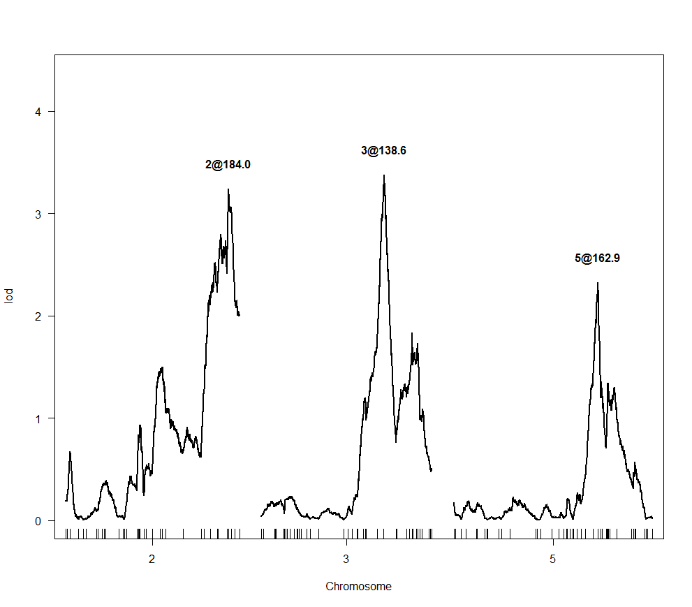


**Chr. 5**

**Chr. 3**

**Chr. 2**

**Chr. 3**

**Chr. 2**

**LOD**

**Figure S5. LOD profiles for QTLs underlying the accumulation of selected isoprenoids in the AI-RILs**: (A) polyprenols, (B) dolichols, (C) chlorophylls, and (D) carotenoids (see Materials and Methods).


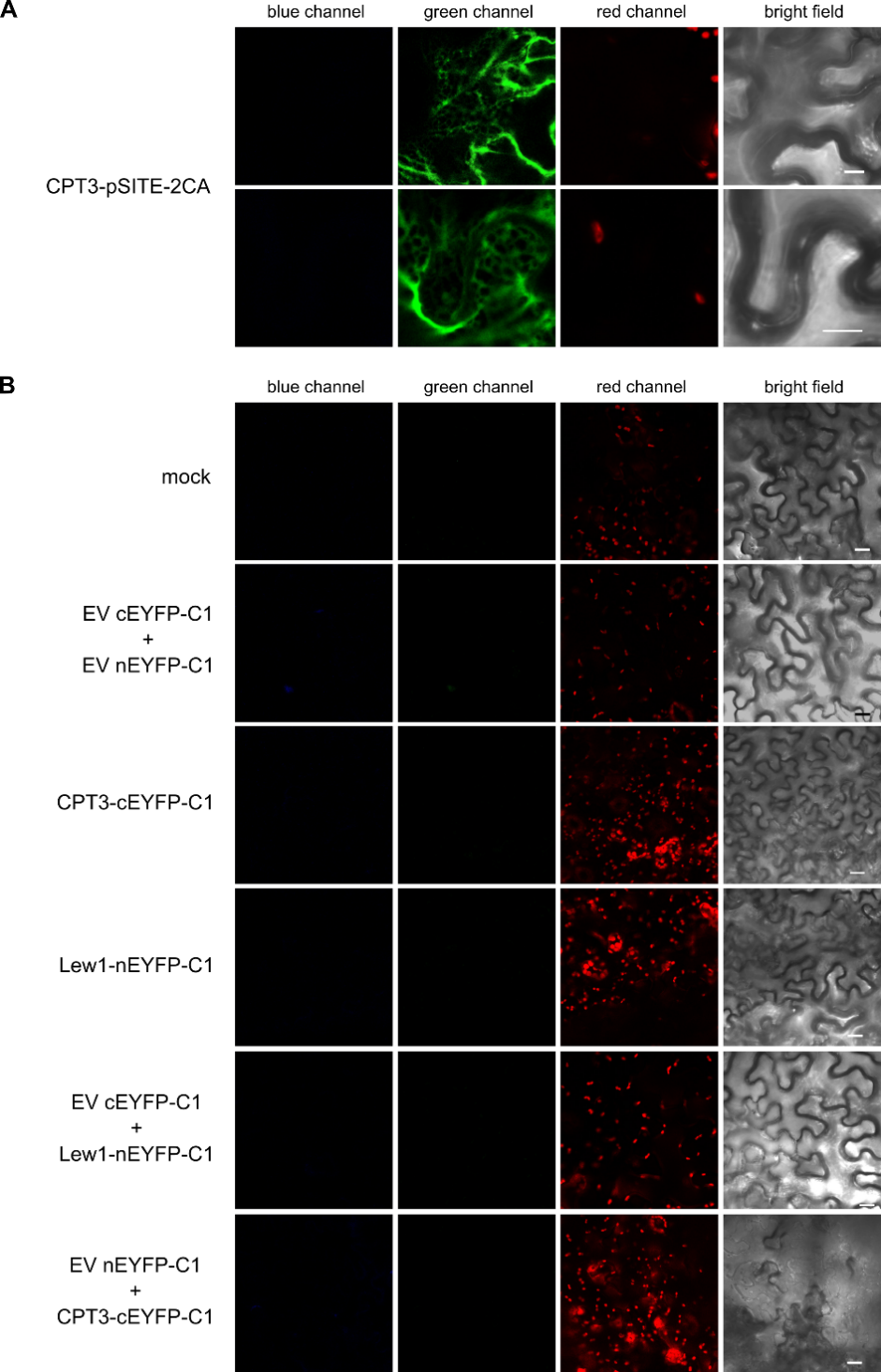


**Figure S6. Subcellular localization of CPT3 in confocal microscopy (A) and analysis of CPT3 and Lew1 protein–protein interaction - negative controls of BiFC assay (B).**
(A) *Nicotiana benthamiana* leaves were transiently transformed with CPT3 fused at the N-terminus to GFP. Green fluorescence attributable to GFP and recorded only in the green channel was observed in the structures corresponding to the ER membranes. The chlorophyll autofluorescence was observed in the red channel. Scale bars = 10 mm. Co-localization of fluorescence signals of CPT3-GFP and the compartmental marker ER-CFP is presented in Figure 2D.

(B) Mock - when tobacco leaves were infiltrated only with infiltration buffer, no green fluorescence attributed to EYFP was observed. Co-transformation of empty vectors EV cEYFP- and EV nEYFP- did not show green fluorescence of EYFP. Single transformation with CPT3-cEYFP-C1 or LEW1-nEYFP-C1 did not show green fluorescence of EYFP. Co-transformation of LEW1-nEYFP-C1 or CPT3-cEYFP-C1 with the empty vectors EV cEYFP- or EV nEYFP- did not show interaction. Red autofluorescence of chlorophyll was observed for all studied control variants. Scale bars = 20 mm.

**Tocopherols**

**Carotenoids**

**Chlorophylls**

**Polyprenols**


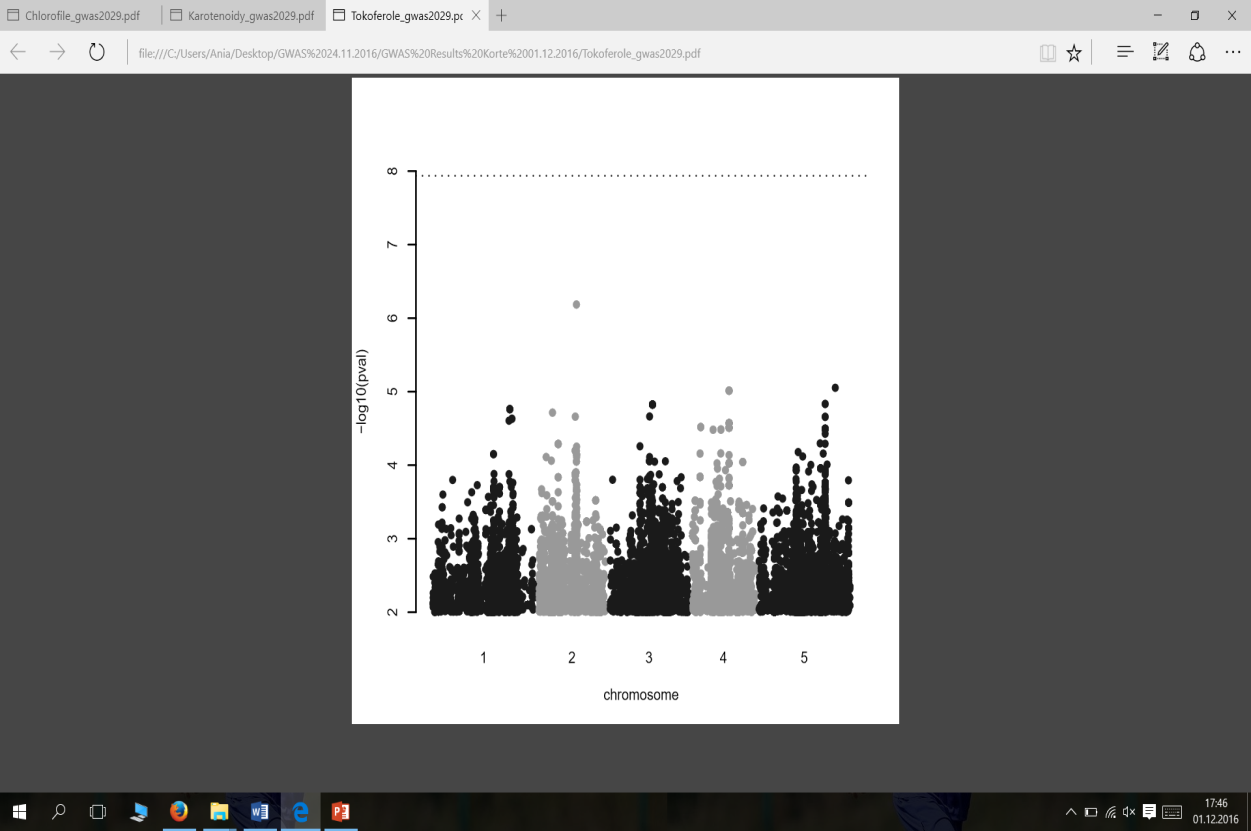

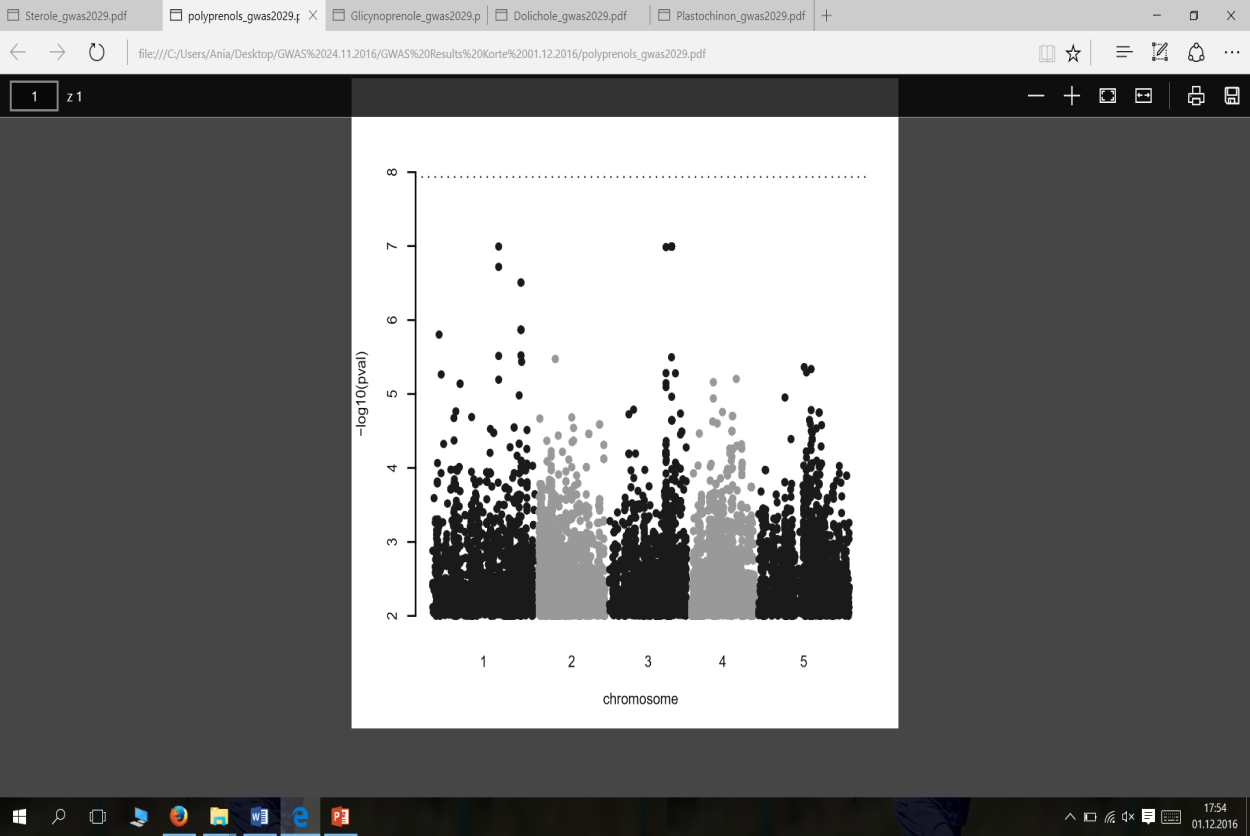


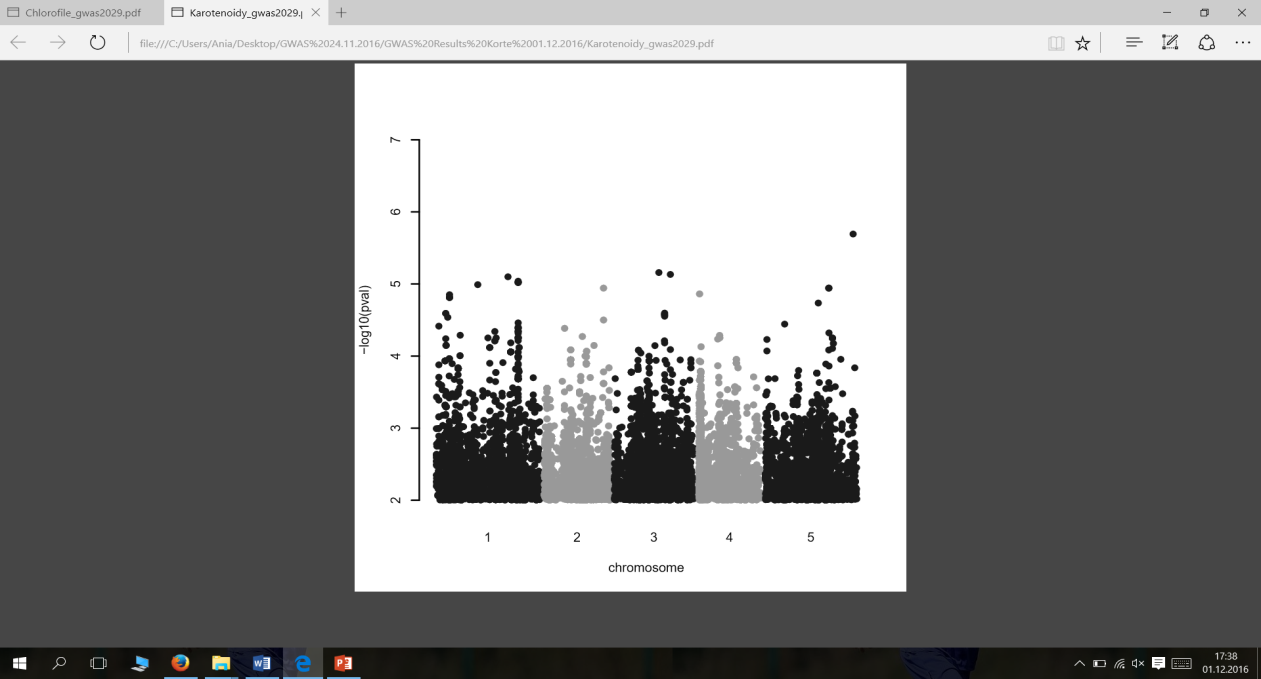

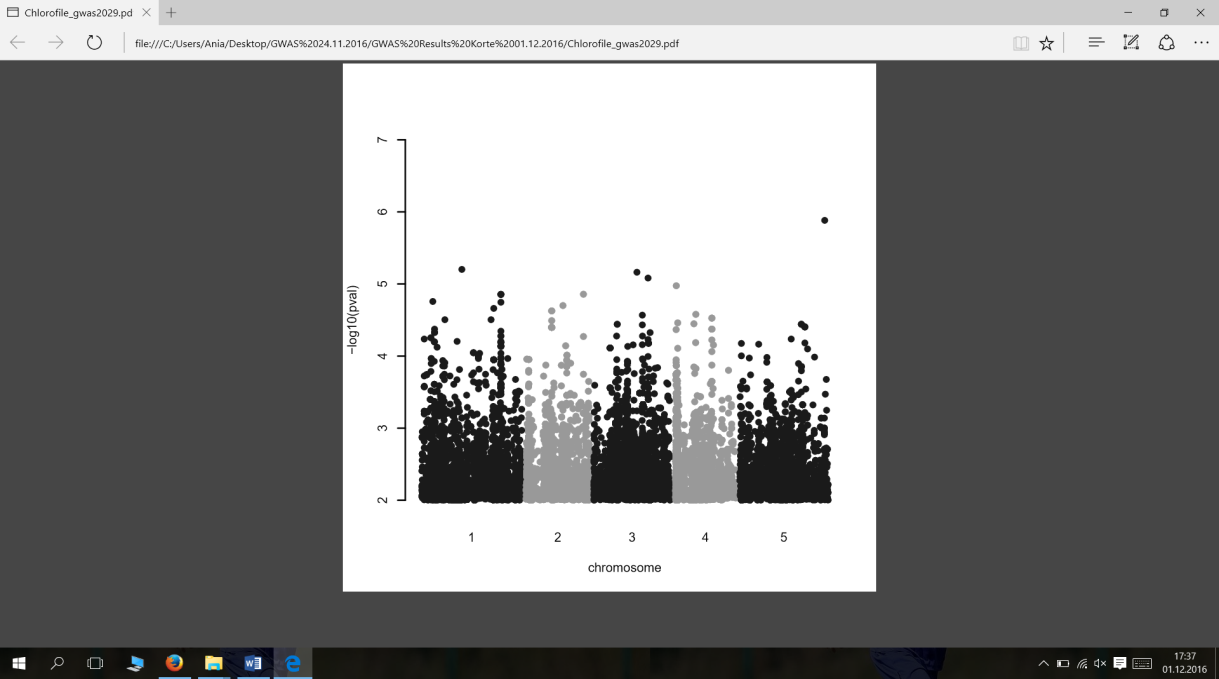


**Figure S7.** **Manhattan plot of genome-wide association results for polyprenols, chlorophylls and tocopherols.** The dotted horizontal lines indicate a significance level of 0.05 after Bonferroni correction for multiple testing. See Material and Methods.

**Commentary note to Figure S7.**

Analysis of associations revealed that in addition to Dols several SNPs were detected also for plastoquinone and phytosterols. For plastoquinone, 26 SNPs, spread across 7 distinct genomic regions, have been found. The most significant SNP is located at chromosome 1 at position 19,545,459 and is identical to the one reported for Dol. Despite the fact that other significant SNPs are spread over 3 different chromosomes, they are all in linkage disequilibrium (LD) with each other, indicating only one independent association. And indeed, if the lead SNP is added as a cofactor to the model (Segura et al., 2012), none of the remaining SNPs stays above the threshold, indicating only one causative association. It is noteworthy that many of these other SNPs are directly located in transposable elements. For phytosterols, 10 SNPs at 5 distinct genetic regions showed significant associations. One of these is again the same SNP that has been reported above for plastoquinone and Dols. Interestingly, this polymorphism does not show the strongest association with phytosterols, but three other sequence variants, located around 19.67 Mb on chromosome 3, are in perfect LD and show a stronger association. These polymorphisms are located between AT3G53040, encoding a LEA protein, and AT3G53050, which encodes an enzyme involved in hydrolyzing *O*-glycosyl compounds.


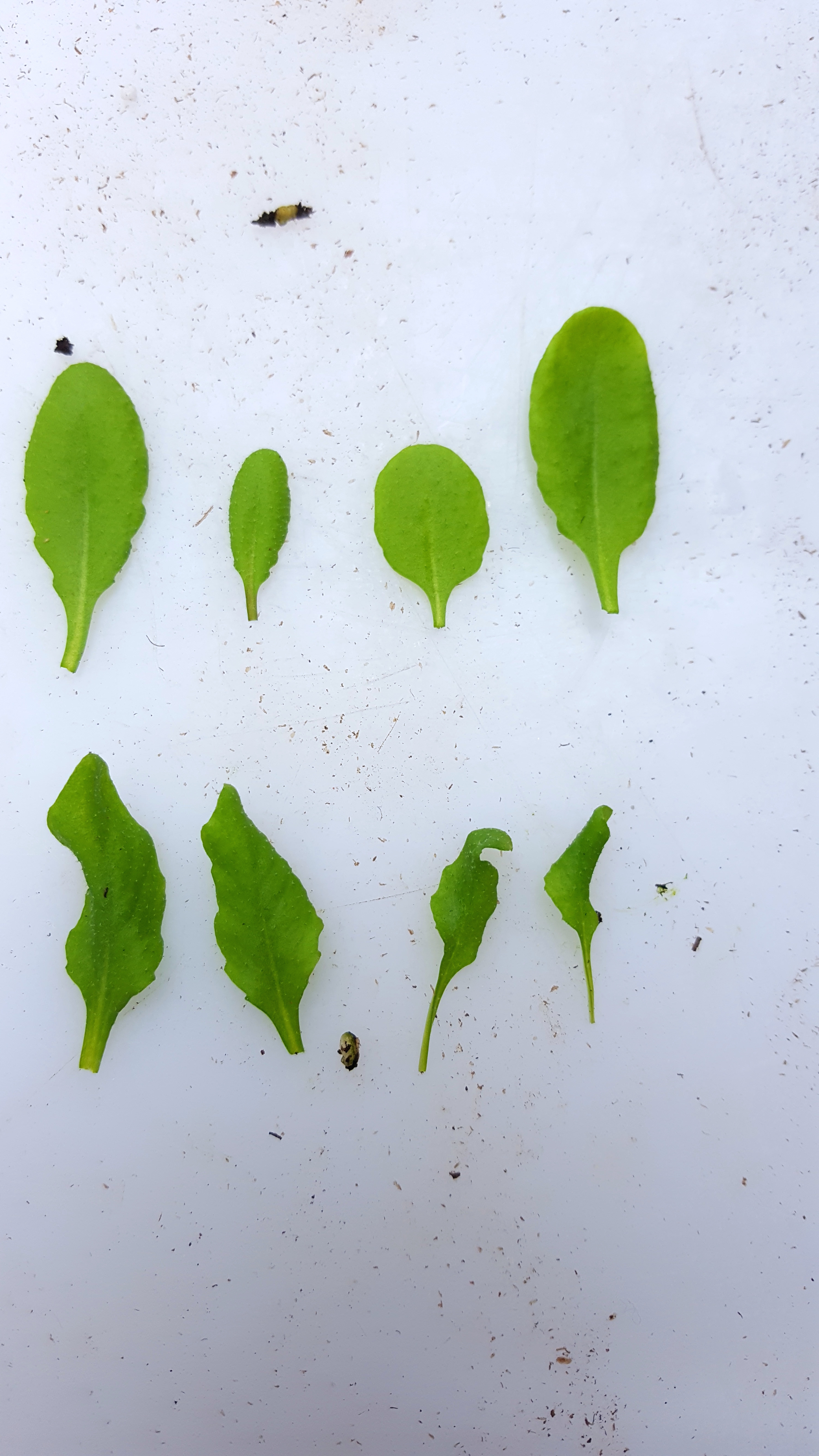


**Col-0**

(wild-type plants)

**AT1G52460-deficient plants**

(heterozygous mutant)

**Figure S8. The phenotypic appearance of 4-week-old detached leaves of AT1G52460-deficient line** (SALK_066806, *α/β-hydrolase*, heterozygous mutant) and wild-type (Col-0) plants grown in soil.

**(M)**

**(E)**


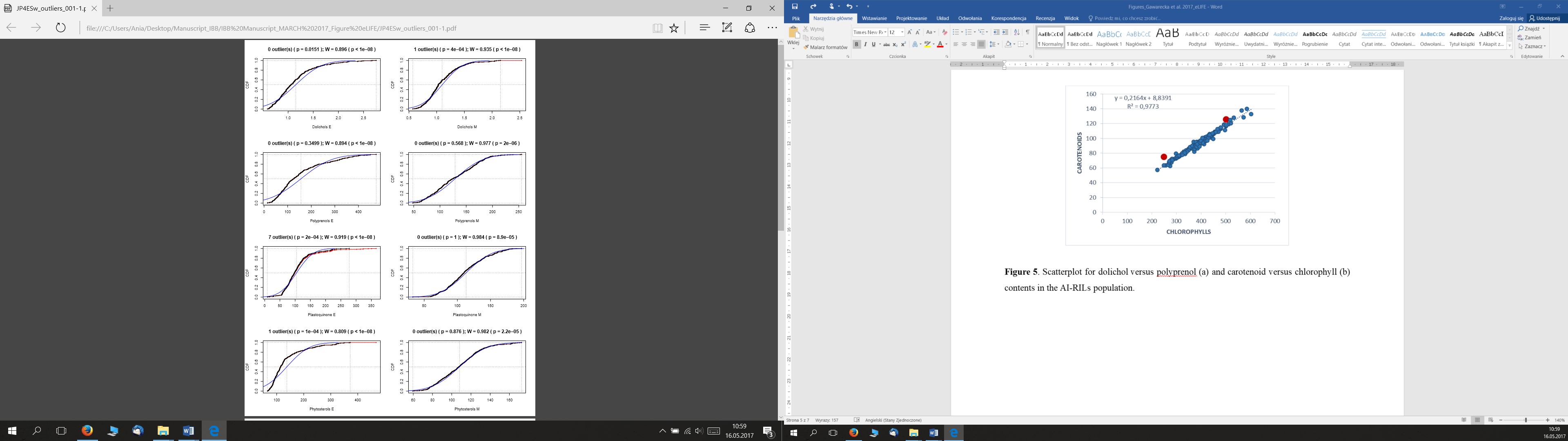


**(M)**

**(E)**


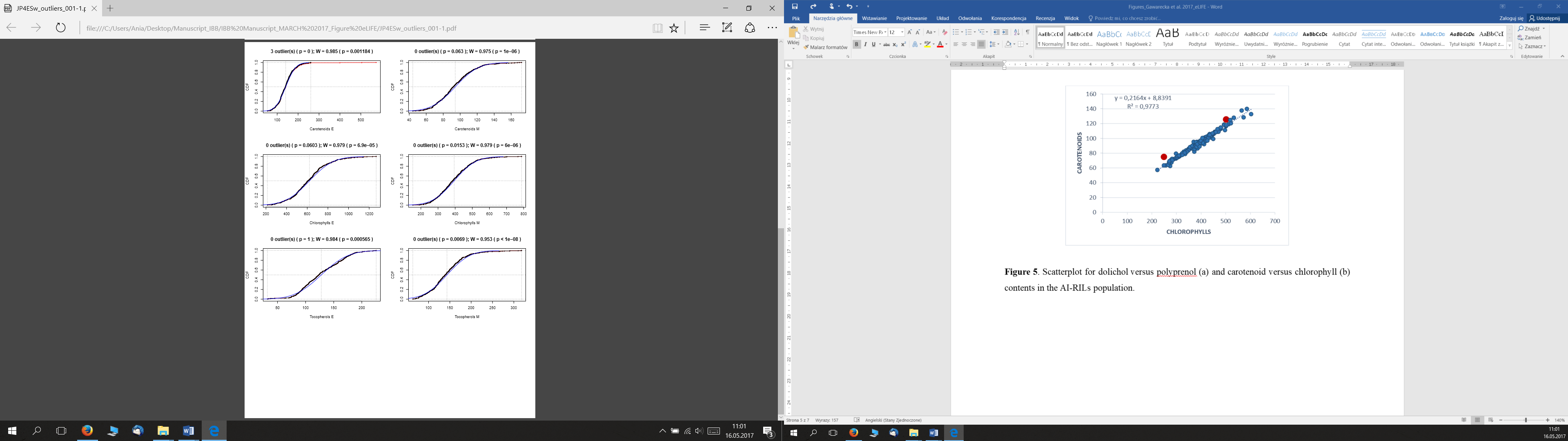


**Figure S9. Cumulative distributions (CDF) of the content of seven studied metabolites analyzed in the seedlings of Arabidopsis accessions (E) and AI-RILs (M) (left and right column, respectively).** Each set of data, presented in a single panel, was analyzed to check for the presence of outliers (Grubbs test at significance level α=0.001), and for normal distribution of the data filtered out of outliers (Shapiro-Wilk test). Red markers follow original distributions, while black ones show the same data with outliers removed. Blue lines represent the CDF expected for the normal distribution. Short statistics for Grubbs (G, p) and Shapiro-Wilk (W, p) tests are shown above each panel. See Material and Methods.


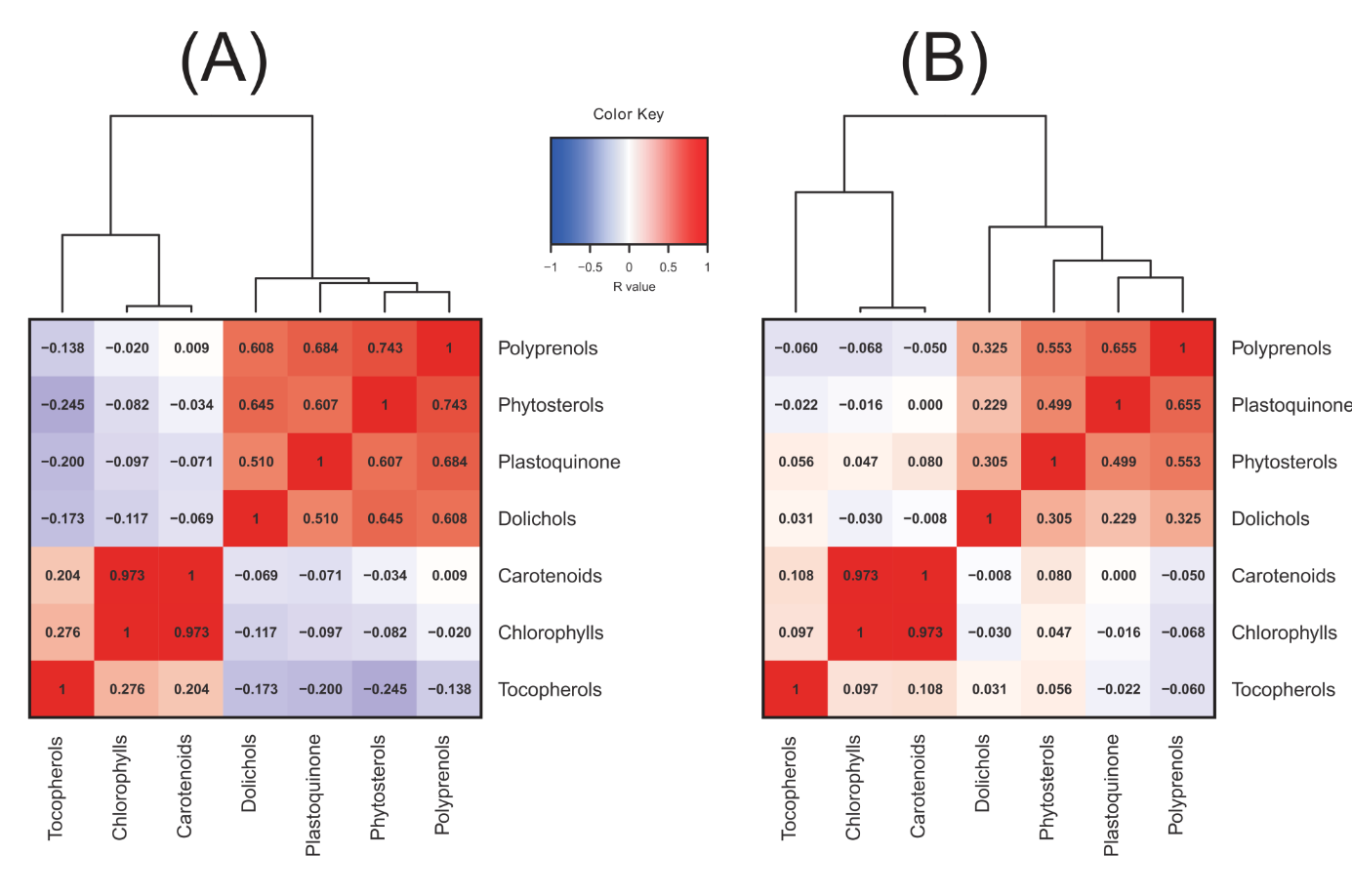


**Figure S10. Dendrograms and corresponding heatmaps calculated for the accessions (A) and the mapping population (B).** Hierarchical cluster analysis was performed for correlation matrixes (built of Spearman’s rank correlation coefficients, R) using the Lance–Williams dissimilarity update formula according to Ward’s clustering algorithm (Ward J, 1963) (see Materials and methods).

**Commentary notes to Figure S10.**

Besides analysis of correlations the same data were employed to analyze hierarchical clustering, in which the correlation matrix was used as a measure of the distance between metabolites in the natural accessions and the mapping population. This clearly showed relationships between metabolite levels (Figure S10), which might reflect coupling(s) in their biosynthetic pathways (Figure 6). Thus, chlorophylls and carotenoids were the most closely related compounds (Figure S10), while phytosterols, plastoquinone, and Prens formed a separate cluster, which was also attracting the Dol cluster. The Dol cluster was, however, much more distant from the three other metabolites. The most distant cluster was comprised of tocopherols and it did not seem to correlate significantly with any other metabolite. A small disagreement between the trees deduced for the natural accessions or for the EstC mapping population was found concerning the location of phytosterols and plastoquinone vs. Prens (Figure 5B and Figure S10).

**Trait correlations**

Strong correlations between the levels of particular metabolites (Figure 5 and Figure S10) probably mirror common mechanisms responsible for their formation. Despite the fact that all studied metabolites are derived from the isoprenoid pathway, their clustering reflects the complexity of this pathway (Figure 6). Thus, the tight (‘perfect’) association of carotenoids and chlorophylls is in agreement with their biosynthetic origin from a common isoprenoid precursor, geranylgeranyl diphosphate (GGPP), and the plastidial localization of their biosynthesis (Figure 6). Moreover, IPP molecules required for the synthesis of carotenoids, chlorophylls, and plastoquinone are thought to be derived mostly from the methylerythritol phosphate (MEP) pathway and several, but not all, steps of their synthesis are located in plastids too. Prens seem to cluster more closely with plastoquinone than with Dols (Figure S10), despite the high similarity of Pren and Dol structure. Interestingly, a growing body of information suggests that the biosynthetic routes for Prens and Dols in plants are different. Prens are synthesized in plastids (Akhtar et al., 2017), probably from MEP-derived IPP with GGPP used as intermediate, while Dols are synthesized consecutively in plastids and in the cytoplasm (Skorupinska-Tudek et al., 2008; Jozwiak et al., 2017) with the concomitant involvement of the MEP and MVA pathways and involvement of FPP (Figure S1, Figure 6). Thus, the assignment of Prens and Dols to distinct clusters and the calculated dendrogram are in line with the described above differences in their biosynthetic routes. In addition to the coupling of the biosynthetic pathways observed trait correlations, shown by the heatmaps, might also suggest their coordinated involvement in various cellular processes - intimately linked function of carotenoids and chlorophylls in photosynthesis is a good example of such phenomenon. Further studies are needed, however, to get deeper insight into these aspects of plant physiology.

**Table S1. Characteristics of the detected QTLs underlying polyprenol (Pren), dolichol (Dol), chlorophyll and carotenoid accumulation in the AI-RIL population** (see Materials and Methods).

| **Trait** | **QTL** | **Chr^a^** | **LOD score** | **Peak^b^ (cM)** | **Confidence interval^c^ (cM)** | **Confidence**  **interval (bp)** | **PVE^d^  (%)** | **Number of genes** |
| --- | --- | --- | --- | --- | --- | --- | --- | --- |
| Dolichols | DOL1 | 2 | 5.86 | 67.9 | 64.8 – 74.4 | 7237666 –  8146712 | 16.88 | 308 |
| Polyprenols | PRE1 | 5 | 3.506 | 132.1 | 123.2 – 138.3 | 13814976 – 15171769 | 9.60 | 375 |
|  | PRE2 | 5 | 4.451 | 169.3 | 166.5 – 170.7 | 18065657 – 19123615 | 12.38 | 334 |
|  | PRE3 | 5 | 3.921 | 173.2 | 172.1 – 174.1 | 19123616 – 19715719 | 10.81 | 239 |
| Chlorophylls | CHL1 | 2 | 3.838 | 178.6 | 160.8 – 191.6 | 15251663 – 18694069 | 10.78 | 1370 |
|  | CHL2 | 3 | 1.937 | 137.9 | 111.6 – 188.1 | 11208231 – 22787413 | 5.28 | 3658 |
| Carotenoids | CAR1 | 2 | 3.241 | 184 | 159.3 – 196.5 | 15251663 – 19601673 | 8.20 | 1745 |
|  | CAR2 | 3 | 3.373 | 138.6 | 131.3 – 145.6 | 16551391 – 18590589 | 8.55 | 698 |
|  | CAR3 | 5 | 2.327 | 162.9 | 151.3 – 187.2 | 16259147 – 21318882 | 5.80 | 1778 |
| ^a^ Chromosome number; ^b^ Position of peak; ^c^ 1-LOD support interval; ^d^ Percentage of phenotypic variance explained by the QTL (PVE). | | | | | | | | |

**Table S2**. **Selection of candidate genes from chosen QTL intervals.**

We selected one QTL for Dol (DOL1) and three QTLs associated with Pren accumulation (PRE1, PRE2, PRE3) for further *in silico* analyses. The selected intervals were characterized by the highest percentage of phenotypic variance explained by each QTL and the highest LOD (logarithm of the odds) score values linked with the lowest number of loci (Table S1). The positional candidate genes within QTL confidence intervals were extracted from the Araport11 Annotation (www.araport.org). Firstly, we checked the annotated functions for all genes located in the selected QTL intervals by analyzing available databases and literature data for the isoprenoid biosynthetic pathways (TAIR, http://www.arabidopsis.org; PubMed, https://www.ncbi.nlm.nih.gov/pubmed). In this way, we obtained lists of candidate genes putatively considered to be involved in Pren and Dol biosynthesis/accumulation (Table S2 and Table S3). Subsequently, we performed *in silico* analyses focused on tissue distribution and expression levels of the selected genes using data from the Arabidopsis eFP Browser 2.0 database (http://bar.utoronto.ca). This procedure allowed us to generate four sets of genes ‒ three for Pren (Table S2) and one for Dol (Table S3). Detailed SNP analyses of At2G17570 (*CPT3*), AT1G52450 (*UCHs*), and AT1G52460 (*ABH*) sequences (Table S8) in the Arabidopsis population were extracted from the Arabidopsis 1001 genomes data using a custom R script.

**Candidate genes potentially involved in polyprenol accumulation, selected from the mapped intervals (a) PRE1, (b) PRE2 and (c) PRE3.**

Among several putative candidate genes detected in the intervals for Prenols of special interest seem: **(1)** At5G45940 encoding Nudix hydrolase 11 (Kupke, Caparrós-Martín, Malquichagua Salazar, & Culiáñez-Macià, 2009) with putative IPP isomerase activity since isomerization of IPP towards dimethylallyl diphosphate is required to initiate the synthesis of a polyisoprenoid chain; **(2)** At5G47080 encoding the protein kinase CKB1; **(3)** At5G36001 and At5G45790 encoding protein ubiquitination related proteins; **(4)** At5G47980 encoding an acyltransferase; **(5)** At5G36110, At5G4800, At545040 encoding oxidoreductases; **(6)** At5G46760 encoding the transcription factor MYC3; **(7)** AT5G45710 encoding a heat shock related protein; **(8)** At5G48490 encoding a protein implicated in lipid transport and **(9)** At5G48110 tentatively assigned as a terpenoid cyclase/protein prenyltransferase superfamily protein (Araport project).

| 1. *Candidate genes selected from the QTL interval (PRE1) on chromosome 5* | | |
| --- | --- | --- |
| **LOCUS** | **DESCRIPTION (TAIR)** | **FUNCTION** |
| AT5G35920 | a cytochrome P450 pseudogene | **oxidoreductases** |
| AT5G36001 | RING/U-box superfamily protein; | **ligase**; HMGR degradation via ubiquitination |
| AT5G36110 | CYP716A1:member of CYP716A; | **oxidoreductases**; polyisoprenoid oxydation |
| AT5G36140 | member of CYP716A; | **oxidoreductases** |
| AT5G36220 | member of CYP81D family of cytochrome p450s. This gene was originally called CYP91A1 | **oxidoreductases** |

| 1. *Candidate genes selected from the QTL interval (PRE2) on chromosome 5* | | |
| --- | --- | --- |
| **LOCUS** | **DESCRIPTION (TAIR)** | **FUNCTION** |
| AT5G45040 | CYTC6A; | **oxidoreductases**; Encodes a Class I cytochrome c family member |
| AT5G45050 | TTR1 | **transcription factors**; Encodes a member of WRKY factor (Group II-e) that confers resistance to tobacco; regulation of biosynthesis of terpenoid indole alkaloids |
| AT5G45340 | CYP707A3; ABA 8'-hydroxylase | **oxidoreductase** |
| AT5G45420 | maMYB; MYB transcription factor belongs to R2R3-MYB family | **transcription factor** |
| AT5G45430 | Protein kinase superfamily protein; | transferase; protein serine/threonine kinase activity, |
| AT5G45710 | RHA1; member of Heat Stress Transcription Factor (Hsf) family; | **transcription factor** |
| AT5G45750 | RABA1c; RAB GTPase homolog A1C | **hydrolase** |
| AT5G45790 | Ubiquitin carboxyl-terminal hydrolase family protein | **hydrolase** |
| AT5G45890 | SAG12; Senescence-associated gene 12 (SAG12) encoding a cysteine protease influenced by cytokinin, auxin and sugars. | **hydrolase** |
| AT5G45900 | APG7 | **ligase**; Component of autophagy conjugation pathway. Required for proper senescence. Contributes to plant basal immunity towards fungal infection |
| AT5G45940 | NUDT11; | **isomerase**; GGA (geranylgeranylacetone) induces HSP70 through activation of HSF1; potential IPP isomerase |
| AT5G46110 | APE2; Encodes a chloroplast triose phosphate / 3-phosphoglycerate | **transport**; translocator that transports triose phosphates derived from the Calvin cycle in the stroma to the cytosol for use in sucrose synthesis and other biosynthetic processes. |
| AT5G46210 | CUL4 | **ligase**; Arabidopsis CULLIN4 (CUL4) forms an E3 ubiquitin ligase with the CDD complex and a common catalytic subunit RBX1 in mediating light control of development; ubiquitination |
| AT5G46320 | MADS-box proteins | **transcription factor;** lycopene accumulation, fruit ripening |
| AT5G46350 | WRKY8; member of WRKY, Group II-c | **transcription factor** |
| AT5G46660 | C-like zinc finger protein | **transferase** |
| AT5G46740 | UBP21; | **hydrolase**; Encodes a ubiquitin-specific protease |
| AT5G46760 | MYC3 | **transcription factors**; JAZ-interacting transcription factor that act together with MYC2 and MYC4 to activate JA-responses; regulate sequiterpene synthesis |

| 1. *Candidate genes selected from the QTL interval (PRE3) on chromosome 5* | | |
| --- | --- | --- |
| **LOCUS** | **DESCRIPTION (TAIR)** | **FUNCTION** |
| AT5G47080 | CKB1 | **transferase**; Regulatory subunit beta of casein kinase II (CK2) |
| AT5G47230 | ERF5 | **transcription factor**; encodes a member of the ERF (ethylene response factor) subfamily B-3 of ERF/AP2 transcription factor family (ATERF-5) |
| AT5G47240 | NUDT8 | **hydrolase** |
| AT5G47390 | myb-like family protein; | **transcription factor** |
| AT5G47610 | RING/U-box superfamily protein; | **hydrolase**; ubiquitination |
| AT5G47650 | NUDT2 | **hydrolase**; Encodes an ADP-ribose pyrophosphatase that confers enhanced tolerance to oxidative stress |
| AT5G47850 | CCR4 | **transferase;** transcription activator, induced by GERANIOL |
| AT5G47880 | ERF1-1 | **transcription factor**; Encodes a eukaryotic release factor 1 homolog; regulates responses to glucose and phytohormones |
| AT5G47950 | HXXXD-type acyl-transferase family protein | **transferase**; Geraniol /Citronellol Acetyltransferase; potential way of prenol estification |
| AT5G47960 | RABA4C | Encodes a small molecular weight g-protein |
| AT5G47980 | HXXXD-type acyl-transferase family protein; | **transferase**; potential way of prenol estification |
| AT5G48000 | CYP708A2 | **oxidoreductase**; Encodes a member of the CYP708A family of cytochrome P450 enzymes. THAH appears to add a hydroxyl group to the triterpene thalianol |
| AT5G48110 | Terpenoid cyclases/Protein prenyltransferases superfamily protein | **lyases** |
| AT5G48230 | ACAT2 | **transferase**; Encodes an acetoacetyl-CoA thiolase that generates the bulk of the acetoacetyl-CoA |
| AT5G48490 | Bifunctional inhibitor/lipid-transfer protein; | **transport system**; potential way of polyisoprenoid transport |

**Table S3. Candidate genes potentially involved in dolichol accumulation, selected from the mapped QTL interval DOL1.**

Among twenty genes listed in the Table S3, there are three strong putative candidates potentially involved in Dol biosynthesis. Besides the At2G17570 gene encoding CPT3 that is described in detail in the manuscript, two other strong candidates (At2G17370 and At2G18620) are described below.

**At2G17370** encodes a functional HMGR2 reductase (Suzuki et al., 2009). The Arabidopsis genome possesses two genes, *HMGR1* and *-2*, encoding three isoforms of this key regulator of the MVA pathway: HMGR1S has been proposed to have a housekeeping role, whereas HMGR1L and HMGR2 have more specialized functions, possibly required in particular cell types or at specific developmental stages (Suzuki et al., 2009). Since both isoprenoid-generating pathways, the MVA and MEP, contribute to Dol biosynthesis (Jozwiak et al., 2017) a role of HMGR2 in Dol accumulation corroborates with the general concept of the Dol biosynthetic pathway.

**At2G18620** encodes GGPPS2, one of the 12 geranylgeranyl diphosphate synthases predicted in the Arabidopsis genome (Beck et al., 2013). This enzyme might also possibly be involved in Dol biosynthesis as a producer of its all-*trans*-initiator. Although, based on Dol structure (containing two internal *trans* isoprene residues), FPP rather than GGPP seems to serve as the initiator of Dol synthesis, At2G18620 might fulfill this role since its product specificity (FPP vs. GGPP) has not been verified *in vivo*. The list of other candidate genes detected in the QTL region for Dol includes an ubiquitin-conjugating enzyme (At2G16920), protein kinases (e.g., At2G18470 – PERK4 of the ABA signaling pathway), a glycosyltransferase (At2G18560), transcription factors (e.g., At2G16720 – MYB7; At2G18060 – VND1; At2G18160 – bZIP2) and proteins belonging to other functional groups (e.g., At2G18750 – calmodulin binding protein; At2G17870 – cold shock domain protein 3; At2G18480 – carbohydrate transmembrane transporter). For the list of selected genes identified in QTL for Dol see below.

| *Candidate genes selected from the QTL interval (DOL1) on chromosome 2* | | |
| --- | --- | --- |
| **LOCUS** | **DESCRIPTION (TAIR)** | **FUNCTION** |
| AT2G16720 | MYB7: Encodes a member of MYB3R- and R2R3- type MYB- encoding gene | **transcription factor;** regulation of secondary metabolism and cell shape, the enhancement of disease resistance and the response to different stresses. An integrative function of OsMPS in the cross-talk between phytohormones and the environment to regulate adaptive growth; the negative regulator of ABA signaling, and enhances salt tolerance; MYBp had the ability to regulate anthocyanin biosynthesis pathway genes; MYBs integrate diverse nutrient starvation (sugar, P, N) and gibberellin signaling pathways during germination of cereal grains. |
| AT2G16750 | protein kinase activity | protein **kinase** serine/threonine |
| AT2G16920 | UBC23 | **ligase**;ubiquitin-conjugating enzyme |
| AT2G16950 | TRN1:Nuclear import receptor for AtGRP7 | **transport system**; The RNA-binding protein *Arabidopsis thaliana* glycine-rich RNA-binding protein 7 (AtGRP7) regulates the steady-state abundance of numerous target transcripts in *A. thaliana*. GA1 and GA2 transcripts encoding the first enzymes of the gibberellin biosynthetic pathway are expressed at reduced levels in transgenic plants ectopically over-expressing AtGRP7 |
| AT2G17370 | HMG2 | **oxidoreductase** |
| AT2G17390 | AKR2B: High homology to AKR2A | **transcription factor** |
| AT2G17570 | CPT1/CPT3 | **ligase**; Undecaprenyl pyrophosphate synthetase, |
| AT2G17770 | bZIP27 | **transcription factors**,  AREB1/ABF2, AREB2/ABF4, and ABF3, are activated by SNF1-related kinase 2s (SnRK2s) |
| AT2G17870 | CSP3 - COLD SHOCK DOMAIN PROTEIN 3 | **transcription factor**; involved in the acquisition of freezing tolerance |
| AT2G17990 | kinectin-related | **transport system**; kinectin is implicated in shaping, cisternal stacking and cytoskeletal interactions of ER |
| AT2G18060 | VND1; Encodes a NAC-domain; NAC genes involved in transcriptional regulation of leaf senescence, stress tolerance and other developmental stages of cotton | **transcription factor** |
| AT2G18160 | bZIP2 | **transcription factor** |
| AT2G18380 | GATA20 | **transcription factor;** Convergence of auxin and gibberellin signaling on the regulation of the GATA transcription factors GNC and GNL in *Arabidopsis thaliana* |
| AT2G18470 | PERK4 | Proline-rich extensin-like receptor **kinase** 4. Functions at an early stage of ABA signaling |
| AT2G18480 | Major facilitator superfamily protein | **transport system;** carbohydrate transmembrane transporter activity |
| AT2G18530 | Protein kinase superfamily protein | **kinase** |
| AT2G18560 | UDP superfamily protein | Glycosyl**transferase** |
| AT2G18570 | UDP superfamily protein | Glycosyl**transferase** |
| AT2G18620 | Terpenoid synthases superfamily protein | **ligases**; isoprenoid biosynthetic process |
| AT2G18750 | Calmodulin-binding protein | **hydrolase**; calmodulin binding transcription activator (CAMTA) regulates drought response in Arabidopsis |

**Commentary note to Table S3**

In summary, two types of genes were identified by QTL mapping – (i) loci encoding enzymes of the isoprenoid biosynthetic pathways and (ii) loci potentially implicated in this process as regulators or involved in the degradation of polyisoprenoids. These latter genes deserve special attention. Importantly, some of the selected candidate genes underlying the QTL for Dol are highly and/or specifically expressed in roots where Dol is mostly distributed in plant tissues, while the genes underlying the QTL for Pren are expressed mostly in leaves where plants show the highest accumulation of Prens. There are many plausible genes that could underlie the detected QTLs. Candidate genes identified within the QTLs for Dol and Pren accumulation comprise a broad set of genes encoding proteins belonging to various functional classes (proteins involved in ubiquitination, ABA signaling, oxidoreductases, transferases including glycosyltransferases, hydrolases, lyases, isomerases, ligases, chaperones and transcription factors (Table S2 and Table S3). Although products of all these genes could in principle lead to the detected differences in Dol and Pren content in the EstC mapping population, the identification of the causative gene(s) within the detected QTLs could be additionally complicated by the fact that many of these genes are members of gene families with redundant functionalities.

**Table S4. Genetic correlations between metabolite levels.** Estimates for the genetic correlation of the metabolite levels in the natural accessions are shown in the upper triangle together with the estimated standard deviation (± SD). The estimates have been derived from the Multi-trait mixed model. The model did not converge for the analysis of Tocopherols with Chlorophylls and for Tocopherols with Carotenoids (denoted as *nc* and *nd* for *not converged* and *not determined*, respectively). See Materials and methods.

| **Genetic  correlation** | **Chlorophylls** | **Carotenoids** | **Phytosterols** | **Plastoquinone** | **Prenols** | **Dolichols** |
| --- | --- | --- | --- | --- | --- | --- |
| **Tocopherols** | *nc* | *nd* | -0.68 ± 0.29 | -0.75 ± 0.49 | -0.66 ± 0.28 | -0.44 ± 0.30 |
| **Chlorophylls** |  | 0.99 ± 0.01 | -0.39 ± 0.20 | -0.38 ± 0.18 | -0.21 ± 0.15 | -0.30 ± 0.13 |
| **Carotenoids** |  |  | -0.37 ± 0.22 | -0.38 ± 0.20 | -0.18 ± 0.16 | -0.29 ± 0.15 |
| **Phytosterols** |  |  |  | 0.99 ± 0.04 | 0.99 ± 0.02 | 0.94 ± 0.04 |
| **Plastoquinone** |  |  |  |  | 0.99 ± 0.05 | 0.83 ± 0.06 |
| **Prenols** |  |  |  |  |  | 0.89 ± 0.05 |

**Table S5. Segregation of *at1g52460* alleles and seed germination rates of AT1G52460-deficient plants.** Segregation of *at1g52460* alleles of SALK_066806 and GK_823G12 insertion mutants, data were estimated for F1 progeny of self-pollinated heterozygous AT1G52460-deficient mutants. The frequency distribution was analyzed by χ2 test. In parallel seed germination of AT1G52460-deficient plants on solid medium was analyzed too.

| **Segregation of *at1g52460* alleles** | | | | | | **Seed germination** | | |
| --- | --- | --- | --- | --- | --- | --- | --- | --- |
| GK_823G12 n=151 | | | SALK_066806 n=61 | | | seeds | GK_823G12 | SALK_066806 |
| genotype | expected | observed | genotype | expected | observed | sown | 149 | 132 |
| *at1g52460 ^-/-^* | 38 (25%) | - | *at1g52460 ^-/-^* | 15 (25%) | - |  |  |  |
| *at1g52460^+/-^* | 75 (50%) | 110 (73%) | *at1g52460^+/-^* | 31 (50%) | 39 (64%) | germinated | 118 (79%) | 99 (75%) |
| WT | 38 (25%) | 41 (27%) | WT | 15 (25%) | 22 (36%) |  |  |  |
|  | χ ^2^ =2.6 (p>0.1) |  |  | χ ^2^ =0.2 (p>0.65) |  |  |  |  |

**Table S6. Metabolic data-based correlations between metabolite levels.**

Data for the AI-RILs and accessions are shown in the lower and upper triangle, respectively. The statistical significance assessed at p=0.05, 0.01, 0.001 and 0.0001 is denoted by *, **, *** and ****, respectively. See Materials and methods and Source Data 22.

| **Accessions**  **AI-RILs** | **Tocopherols** | **Chlorophylls** | **Carotenoids** | **Phytosterols** | **Plastoquinone** | **Prenols** | **Dolichols** |
| --- | --- | --- | --- | --- | --- | --- | --- |
| Tocopherols |  | 0.276**** | 0.204*** | -0.245**** | -0.200*** | -0.138** | -0.173** |
| Chlorophylls | 0.097* |  | 0.973**** | -0.082 | -0.097 | -0.020 | -0.117* |
| Carotenoids | 0.108* | 0.973**** |  | -0.034 | -0.071 | 0.009 | -0.069 |
| Phytosterols | 0.056 | 0.047 | 0.080 |  | 0.607**** | 0.743**** | 0.645**** |
| Plastoquinone | -0.022 | -0.016 | 0.000 | 0.499**** |  | 0.684**** | 0.510**** |
| Prenols | -0.060 | -0.068 | -0.050 | 0.553**** | 0.655**** |  | 0.608**** |
| Dolichols | 0.031 | -0.030 | -0.008 | 0.305**** | 0.229**** | 0.325**** |  |

**Table S7. Summary of candidate genes involved in accumulation of Dol, plastoquinone, phytosterols and Pren – comparison of QTL and GWAS approaches.**

| Trait | GWAS | | | | | QTL | | | |
| --- | --- | --- | --- | --- | --- | --- | --- | --- | --- |
|  | Location (bp) | Candidate Gene (CG) | Amino Acid exchange | Putative  function | Ref. | Location (bp) | Candidate Gene (CG) | Putative  function | Ref. |
| Dol | Chr 1 19545459 | AT1G52450 first exon | non-synonymous | ubiquitin carboxyl-terminal hydrolase (UCHs) | --- | Chr 2 7237666-8146712 | AT2G17570 | CPT3 | Surmacz & Swiezewska 2011, Kwon et al. 2016 |
|  | Chr 1 19540865 | AT1G52440 3’UTR | --- | an alpha-beta hydrolase | --- |  | AT2G17370 | HMGR2 | Suzuki et al. 2009 |
|  | Chr 3 18558714 18558716 | AT3G50050 exon | non-synonymou, synonymous | Myb77 auxin related TF | Shin et al. 2007 |  | AT2G18620 | GGPPS2 | Beck et al. 2013 |
| Plastoquinone | Chr 1 19545459 | AT1G52450 first exon | non-synonymous | UCHs | --- | --- | | | |
| Phytosterols | Chr 1 19545459 | AT1G52450 first exon | non-synonymous | UCHs | --- | --- | | | |
|  | Chr 3 ~19.67 Mb | between AT3G53040 and AT3G53050 | --- | LEA protein O-glycoside hydrolase | --- |  |  |  |  |
| Pren | --- | | | | | Chr 5 PRE1  13814976-15171769  PRE2 18065657-19123615  PRE3 19123616-19715719 | AT5G36001  AT5G45790  AT5G45940  AT5G46760  AT5G48490 | Proteins ubiquitination related  Nudix hydrolase 11 (putative IPP isomerase)  MYC3 TF  Protein implicated in lipid transport | Kupke et al. 2009 |

**Table S8. Detailed SNP analysis of AT2G17570 (*CPT3*), AT1G52450 (*UCH*) and AT1G52460 (*ABH*) sequences in the Arabidopsis population.** SNPs were extracted from the Arabidopsis 1001 genomes data using a custom R script which can be found at https://github.com/arthurkorte/SNP_extractor.

| **SNPs number** | **AT1G52450** | **AT1G52460** | **AT2G17570** |
| --- | --- | --- | --- |
| total | 298 | 230 | 203 |
| nonsynonymous | 150 | 55 | 30 |
| synonymous | 72 | 15 | 28 |
| start | 0 | 0 | 5 |
| stop | 1 | 2 | 1 |
| low | 83 | 32 | 34 |
| moderate | 150 | 55 | 30 |
| high | 3 | 6 | 1 |
| **length [bp]** | **4582** | **1951** | **2201** |

**Table S9. *Arabidopsis thaliana* accessions used in this study.** All listed accessions were obtained from the stock center NASC (http://arabidopsis.info/).

| **No.** | **Abbreviation (Name)** | **Origin/Location** | **Country** | **No.** | **Abbreviation (Name)** | **Origin/Location** | **Country** |
| --- | --- | --- | --- | --- | --- | --- | --- |
| **1** | Ag-0 | Argentat | France | **61** | Mrk-0 | Markt/Baden | Germany |
| **2** | Alc-0 | Alcalá de Henares | Spain | **62** | Mt-0 | Martuba/Cyrenaika | Libya |
| **3** | An-1 | Antwerpen | Belgium | **63** | Mz-0 | Merzhausen | Germany |
| **4** | Ang-0 | Angleur | Belgium | **64** | N13 | Konchezero | Russia |
| **5** | Bay-0 | Bayreuth | Germany | **65** | NFA-8 | Ascot | UK |
| **6** | Bla-5 | Billaberget | Sweden | **66** | NFA-10 | Ascot | UK |
| **7** | Bil-7 | Billaberget | Sweden | **67** | Na-1 | Nantes | France |
| **8** | Bor-1 | Borky | Slovakia | **68** | Nd-1 | Niederzenz | Germany |
| **9** | Bor-4 | Borky | Slovakia | **69** | Nok-3 | Noordwijk | Netherlands |
| **10** | Br-0 | Brunn | Czech Republic | **70** | Or-0 | Oranienstein | Germany |
| **11** | Bs-1 | Basel | Switzerland | **71** | Ost-0 | Osthammar | Sweden |
| **12** | Buckhorn Pass | Buckhorn Pass | USA | **72** | Oy-0 | Oystese | Norway |
| **13** | Bur-0 | Burren | Ireland | **73** | Pa-1 | Palermo | Italy |
| **14** | C-24 | C24 | Portugal | **74** | Per-1 | Perm | Russia |
| **15** | CIBC-5 | Ascot/Berks | UK | **75** | Petergof | Petergof | Russia |
| **16** | CIBC-17 | Ascot/Berks | UK | **76** | PHW-2 | Florence | Italy |
| **17** | Co |  |  | **77** | Pna-10 | Benton Harbor | USA |
| **18** | Col-0 | Columbia | Poland | **78** | Pna-17 | Benton Harbor | USA |
| **19** | CS22491 | Konchezero | Russia | **79** | Pro-0 | Proaza | Spain |
| **20** | Ct-1 | Catania | Italy | **80** | Pu2-7 | Prudka | Czech Republic |
| **21** | Cvi-0 | Cape Verdi Islands | Cape Verdi Islands | **81** | Pu2-23 | Prudka | Czech Republic |
| **22** | Cvi-1 | Cape Verdi Islands | Cape Verdi Islands | **82** | RRS-7 | North Liberty | USA |
| **23** | Eden-2 | Harnosand area | Sweden | **83** | RRS-10 | North Liberty | USA |
| **24** | Edi-0 | Edinburgh | UK | **84** | Ra-0 | Randan | France |
| **25** | Ei-2 | Eifel | Germany | **85** | Ren-1 | Rennes | France |
| **26** | En-1 | Enkheim/Frankfurt | Germany | **86** | Ren-11 | Rennes | France |
| **27** | Er-0 | Erlangen | Germany | **87** | Rmx-A02 | St. Joseph | USA |
| **28** | Est-1 | Estland | Estonia | **88** | Rmx-A180 | St. Joseph | USA |
| **29** | Fab-2 | Faberget | Sweden | **89** | Rsch-4 | Rschew/Starize | Russia |
| **30** | Fab-4 | Faberget | Sweden | **90** | Shakdara | Pamiro-Alay | Tadjikistan |
| **31** | Fei-0 | St. Maria d. Feira | Portugal | **91** | Sap-0 | Slapy | Czech Republic |
| **32** | Ga-0 | Gabelstein | Germany | **92** | Sav-0 | Slavice | Czech Republic |
| **33** | Gd-1 | Gudow | Germany | **93** | Se-0 | San Eleno | Spain |
| **34** | Ge-0 | Geneva | Switzerland | **94** | Sf-2 | San Feliu | Spain |
| **35** | Got-7 | Goettingen | Germany | **95** | Sorbo | Sorbo | Tadjikistan |
| **36** | Got-22 | Goettingen | Germany | **96** | Sq-1 | Ascot | UK |
| **37** | Gu-0 | Glueckingen | Germany | **97** | Sq-8 | Ascot | UK |
| **38** | Gy-0 | La Miniere | France | **98** | St-0 | Stockholm | Sweden |
| **39** | HR-5 | Ascot/Berks | UK | **99** | Stw-0 | Stobowa/Orel | Russia |
| **40** | HR-10 | Ascot/Berks | UK | **100** | Ta-0 | Tabor | Czech Republic |
| **41** | Hs-0 | Hannover/Stroehen | UK | **101** | Tamm-2 | Tammisari | Finland |
| **42** | Jm-0 | Jamolice | Czech Republic | **102** | Ts-1 | Tossa del Mar | Spain |
| **43** | Ka-0 | Karnten | Austria | **103** | Ts-5 | Tossa del Mar | Spain |
| **44** | Kas-1 | Kashmir | India | **104** | Tsu-1 | Tsushima | Japan |
| **45** | Kin-0 | Kindalville | USA | **105** | Uk-3 | Umkirch | Germany |
| **46** | Knox-10 | Knox | USA | **106** | Ull2-3 | Ullstorp | Sweden |
| **47** | Knox-18 | Knox | USA | **107** | Ull2-5 | Ullstorp | Sweden |
| **48** | Kondara | Kondara | Tadjikistan | **108** | Uod-1 | Ottenhof | Austria |
| **49** | Kz-1 | Karagandy | Kazakhstan | **109** | Uod-7 | Ottenhof | Austria |
| **50** | Kz-9 | Karagandy | Kazakhstan | **110** | Van-0 | Vancouver | Canada |
| **51** | LL-0 | Llagostera | Spain | **111** | Var2-1 | Varhallarna | Sweden |
| **52** | Lc-0 | Loch Ness | UK | **112** | Wa-1 | Warsaw | Poland |
| **53** | Ler-1 | Landsberg | Poland | **113** | Wei-0 |  |  |
| **54** | Lip-0 | Lipowiec/Chrzanow | Poland | **114** | Wil-1 | Wilna | Litauen |
| **55** | Lm-2 | Le Mans | France | **115** | Ws-0 | Wassilewskija | Russia |
| **56** | Lp2-2 | Lipovec | Czech Republic | **116** | Ws-2 | Wassilewskija | Russia |
| **57** | Lp2-6 | Lipovec | Czech Republic | **117** | Wt-5 | Wietze | Germany |
| **58** | Lz-0 | Lezoux | France | **118** | Yo-0 | Yosemite Nat. Park | USA |
| **59** | Lov-1 | Lovvik | Sweden | **119** | Zdr-1 | Zdarec | Czech Republic |
| **60** | Mr-0 | Monte/Tosso | Italy | **120** | Zdr-6 | Zdarec | Czech Republic |

**Table S10**. **Primers used for the construction of the *CPT3* silencing vector, genotyping of AT1G52460 T-DNA insertion mutants and expression studies (see Materials and Methods).**

| Primers used for *CPT3* silencing | |
| --- | --- |
| Primer | Sequence 5’ to 3’ |
| amiR-cpt3_I | GATATCTGTCGAGTTAGAGGCAATCTCTCTTTTGTATTCC |
| amiR-cpt3_II | GATTGCCTCTAACTCGACAGATATCAAAGAGAATCAATGA |
| amiR-cpt3_III | GATTACCTCTAACTCCACAGATTTCACAGGTCGTGATATG |
| amiR-cpt3_IV | GAAATCTGTGGAGTTAGAGGTAATCTACATATATATTCCT |
| Oligo A | CTGCAAGGCGATTAAGTTGGGTAAC |
| Oligo B | GCGGATAACAATTTCACACAGGAAACAG |
| amiR-cpt3-TopoF | CACCCTTGATATCGAATTCCTGCAGC |
| amiR-cpt3-TopoR | TCTAGAACTAGTGGATCCC |
| Genotyping primers | |
| AT1G52460-deficient line | primer sequence |
| SALK_066806F | TGTGTAGGTGCATTCCTTACG |
| SALK_066806R | TTGCAAGCACAACATCGAGC |
| SALK_066806 LBb1 | GCGTGGACCGCTTGCTGCAACT |
| GK_823G12F | ACCTAGGTGTGGTTGCACAT |
| GK_823G12R | GGAGCACGAGAGATGGACATA |
| GK_823G12LB | ATATTGACCATCATACTCATTGC |
| qPCR primers | |
| locus / gene | primer sequence |
| AT2G17570 / *CPT3*F | GCGCTTATGTCGATGCTG |
| AT2G17570 / *CPT3*R | CAGACTCAACCTCCTCAGG |
| AT1G52440 / *ABH* putativeF | GCGATTCTGTTCCTCACCCC |
| AT1G52440 / *ABH* putativeR | AGCTGGAGCTTCTCTTCCAAC |
| AT1G52450 / *UCH* putativeF | GCGGTCACCTACAAGGTTCT |
| AT1G52450 / *UCH* putativeR | CTGCATCTGCCTTGGCTTTG |
| AT1G52460 / *ABH* putativeF | CTCATCAAAACATGGCGGCT |
| AT1G52460 / *ABH* putativeR | GTTGGGAGCACGAGAGATGG |
| PP2AA3F | GCGGTTGTGGAGAACATGATACG |
| PP2AA3R | GAACCAAACACAATTCGTTGCTG |
| SANDF | CAAGGCAGGAAATCACCAGGTTG |
| SANDR | CTGTACAGCTGATGCAGACCAG |

**Supplementary Methods - Quantitative analysis of isoprenoids**

Isoprenoid compounds were isolated and quantified as described earlier. Some modifications are indicated below.

***Prens, Dols, and phytosterols****:* analyses were performed as described earlier with modifications (Gawarecka and Swiezewska 2014). Briefly, 3 g of fresh seedlings, supplemented with internal standards of Pren-14 (15 μg) and cholestenol (10 μg), were homogenized in 20 ml of chloroform/methanol solution (1/1, v/v) and extracted for 24 h at 25 °C, lipids were subjected to alkaline hydrolysis, purified on silica gel columns, dissolved in isopropanol (final concentration 6 mg per 1 ml) and stored at -20 °C until used. ***Plastoquinone****:* 0.5 g of seedlings was used. Isolation procedure was as described above, but the hydrolysis step was omitted, and the samples were protected from light. ***Chlorophylls and carotenoids****:* 0.2 g of seedlings was homogenized in acetone, extracted for 24 h at 25 °C, centrifuged (2500 × *g*) and the supernatant was directly subjected to spectrophotometric analyses. All isolation steps were performed in darkness. ***Tocopherols****:* 3 g of seedlings were homogenized in 6 ml ethanol and extracted for 24 h at 25 °C, the sample was supplemented with 4 ml of water and 3 ml of a mixture of hexane/dichloromethane (9/1, v/v) to separate the phases. Water phase was re-extracted 3 times with 3 ml of hexane/dichloromethane, organic phases were pooled and evaporated, lipids were dissolved in 8 ml of dichloromethane and analyzed directly. During the preparation, samples were protected from light.

**HPLC/UV analyses of polyisoprenoids and plastoquinone**

HPLC/UV analyses of ***polyisoprenoids*** were performed as described earlier (Gawarecka and Swiezewska, 2014) with modifications. Briefly, a Waters dual λ absorbance detector and a 4.6 × 75 mm ZORBAX XBD-C18 (3.5 µm) column (Agilent, USA) were used. The applied solvent system was (A) methanol/water (9/1, v/v), (B) methanol/hexane/propan-2-ol (2/1/1, v/v/v) and a gradient program was from 100 – 35% A for 3 min, 35 – 0% A for 7 min, 100% B for 8 min. Qualitative analyses were performed using external standards – mixtures of Prens (Pren-9, -11, …, -23, -25) and Dols (Dol-16 to -21) – while quantitative analyses were performed using the internal standard, Prenol-14. All standards were from the Collection of Polyprenols, IBB PAS, Warsaw, Poland. HPLC/UV analyses of ***plastoquinone*** were performed using the above protocol with a slightly modified gradient: 100 – 35% A for 3 min, 35 – 0% A for 7 min, 100% B for 5 min.

**GC/FID analysis of phytosterols and tocopherols**

GC analysis was performed employing an Agilent Technologies, 7890A apparatus equipped with a split/splitless injector and an FID detector with an HP-5 column (J & W Scientific Columns, Agilent Technologies) 30 m × 0.32 mm and 0.25 µm film thickness. ***Phytosterols*** were analyzed as described previously (Jozwiak et al., 2013). Signals were identified by comparison with external standards (Sigma-Aldrich-Fluka, Poznan). The following compounds were identified in plant samples: campesterol, stigmasterol, β-sitosterol, stigmast-4,22-dien-3one, stigmast-4en-3-one, brassicasterol, β-sitostanol, cholesterol. Total content of phytosterols was used for further analyses. ***Tocopherols*** were analyzed as described previously (Kadioglu, Demirkaya, & Demirkaya, 2009). Signals of tocopherol α, δ and γ were identified by comparison with external standards (a kind gift of Prof. Gustav Dallner, University of Stockholm). Total content of tocopherols was used for further analyses.

**Spectrophotometric analyses of chlorophylls and carotenoids**

***Chlorophylls and carotenoids*** were analyzed as described earlier (Lichtenthaler and Buschman, 2001). All analyses were performed in triplicate (three independent biological replicates). The amounts of all isoprenoid compounds were expressed as μg per g of fresh weight.

**REFERENCES**

Beck, G., Coman, D., Herren, E., Ruiz-Sola, M.A., Rodríguez-Concepción, M., Gruissem, W. & Vranová, E. (2013) Characterization of the GGPP synthase gene family in Arabidopsis thaliana. *Plant Molecular Biology*, *82*, 393–416.

Gawarecka, K. & Swiezewska, E. (2014) Analysis of plant polyisoprenoids. *Methods in Molecular Biology*, *1153*, 135–147.

Kadioglu, Y., Demirkaya, F. & Demirkaya, A.K. (2009) Quantitative determination of underivatized α‐tocopherol in cow milk, vitamin and multivitamin drugs by GC‐FID. *Chromatographia*, *70*, 665–670.

Lichtenthaler, H.K. & Buschmann, C. (2001) Chlorophylls and carotenoids: measurement and characterization by UV/VIS spectroscopy. *Current Protocols in Food Analytical Chemistry*, *1*, F4.3.1–F4.3.8.

Segura, V., Vilhjálmsson, B.J., Platt, A., Korte, A., Seren, Ü., Long, Q., Nordborg, M. (2012) An efficient multi-locus mixed model approach for genome-wide association studies in structured populations. *Nature Genetics*, *44*, 825–830.

Shin, R., Burch, A.Y., Huppert, K.A., Tiwari, S.B., Murphy, A.S., Guilfoyle, T.J., Schachtman, D.P. (2007) The Arabidopsis transcription factor MYB77 modulates auxin signal transduction. *Plant Cell*, *19*, 2440–2453.

Skorupinska-Tudek, K., Poznanski, J., Wojcik, J., Bienkowski, T., Szostkiewicz, I. & Zelman-Femiak, M., Bajda, A., Chojnacki, T., Olszowska, O., Grunler, J., Meyer, O., Rohmer, M., Danikiewicz, W. & Swiezewska, E. (2008) Contribution of the mevalonate and methylerythritol phosphate pathways to the biosynthesis of dolichols in plants. *Journal of Biological Chemistry*, *283*, 21024–21035.

Suzuki, M., Nakagawa, S., Kamide, Y., Kobayashi, K., Ohyama, K., Hashinokuchi, H., Kiuchi, R., Saito, K., Muranaka, T. & Nagata N. (2009) Complete blockage of the mevalonate pathway results in male gametophyte lethality. *Journal of Experimental Botany*, *60*, 2055–2064.
